# Long non-coding RNA Linc-RAM enhances myogenic differentiation by interacting with MyoD

**DOI:** 10.1038/ncomms14016

**Published:** 2017-01-16

**Authors:** Xiaohua Yu, Yong Zhang, Tingting Li, Zhao Ma, Haixue Jia, Qian Chen, Yixia Zhao, Lili Zhai, Ran Zhong, Changyin Li, Xiaoting Zou, Jiao Meng, Antony K. Chen, Pier Lorenzo Puri, Meihong Chen, Dahai Zhu

**Affiliations:** 1 The State Key Laboratory of Medical Molecular Biology, Institute of Basic Medical Sciences, Chinese Academy of Medical Sciences and Department of Biochemistry and Molecular Biology, School of Basic Medicine, Peking Union Medical College, 5 Dong Dan San Tiao, Beijing 100005, China; 2 Department of Biomedical Informatics, School of Basic Medical Sciences, Peking University Health Science Center, Beijing 100191, China; 3 Department of Biomedical Engineering, College of Engineering, Peking University, Beijing 100871, China; 4 Developmental Aging and Regeneration Program, Sanford-Burnham-Prebys Medical Discovery Institute, La Jolla, California 92037, USA; 5 Department of Epigenetics and Regenerative Medicine, IRCCS Fondazione Santa Lucia, Rome 00161, Italy

## Abstract

Long non-coding RNAs (lncRNAs) are important regulators of diverse biological processes. Here we report on functional identification and characterization of a novel long intergenic non-coding RNA with MyoD-regulated and skeletal muscle-restricted expression that promotes the activation of the myogenic program, and is therefore termed Linc-RAM (Linc-RNA Activator of Myogenesis). *Linc-RAM* is transcribed from an intergenic region of myogenic cells and its expression is upregulated during myogenesis. Notably, *in vivo* functional studies show that *Linc-RAM* knockout mice display impaired muscle regeneration due to the differentiation defect of satellite cells. Mechanistically, Linc-RAM regulates expression of myogenic genes by directly binding MyoD, which in turn promotes the assembly of the MyoD–Baf60c–Brg1 complex on the regulatory elements of target genes. Collectively, our findings reveal the functional role and molecular mechanism of a lineage-specific Linc-RAM as a regulatory lncRNA required for tissues-specific chromatin remodelling and gene expression.

## Abstract (web summary)

Long non-coding RNAs are important regulators of many diverse biological processes. Here the authors describe Linc-RAM, which regulates myogenesis by binding MyoD and promoting the assembly of the MyoD–Baf60c–Brg1 complex at target genes.

An increasing number of long (>200 nucleotides) non-coding RNAs (lncRNAs) have been identified as recently annotated^1^. Interestingly, some of these lncRNAs exhibit cell-type-specific expression patterns and have been shown to play pivotal roles in developmental processes, including cell fate determination, cellular differentiation, regulation of the cell cycle and proliferation, apoptosis and aging^2^. They have also been implicated in regulation of the pluripotent state and initiation of differentiation programs in stem cells^3^. A recent study employing an lncRNAs knockout (KO) mouse approach has provided further support for the functional relevance of lncRNAs in regulating the cell differentiation and development, showing that individual KO of 18 different lncRNAs leads to a variety of developmental defects affecting diverse organs, including the lung, gastrointestinal tract and heart^4^. Moreover, mechanistic studies of lncRNAs functions during the cell differentiation and development have revealed that most lncRNAs function by guiding chromatin modifiers and epigenetic regulators to specific genomic loci^5^,^6^. In most cases, this is achieved by recruiting repressive modifiers, such as DNA methyltransferase 3, polycomb repressive complexes^7^ or histone H3 lysine 9 (H3K9) methyltransferases^8^, although transcriptional activation has also been demonstrated through recruitment of the histone H3K4 methyltransferase MLL1 complex^9^,^10^. A nuclear lncRNAs, known as D4Z4 binding element-transcript (DBE-T), which links copy number variation to a polycomb/trithorax epigenetic switch, has been implicated in facioscapulohumeral muscular dystrophy^11^.

Myogenesis is a highly coordinated developmental process. Myogenic cell specification and differentiation is determined by the master transcriptional regulatory factor MyoD (myogenic differentiation) in concert with other myogenic regulatory factors (MRFs), such as the muscle bHLH proteins Myf5, myogenin (MyoG) and MRF4, and with the MEF2 family members^12^,^13^,^14^. MyoD and Myf5, which are expressed at the time of myogenic specification, initiate muscle gene expression by virtue of their ability to remodel chromatin at previously silent target loci^15^ that is conferred by the association with chromatin-modifying enzymes, such as histone acetyltransferases, methyltransferases and the ATPase-dependent chromatin-remodelling SWItch/Sucrose NonFermentable (SWI/SNF) complex^16^. Although recent studies have revealed that the association between MRFs and these ‘chromatin modifiers' is directed by extracellular signal-activated pathways, such as p38 and AKT signalling^17^,^18^,^19^,^20^, the identity of potential mediators of these interactions is still missing.

The cell-type-specific expression pattern of lncRNAs and their proposed function as ‘chromatin modifiers' at specific genomic loci, predict that lncRNAs facilitate association of tissue-specific transcriptional activators and general co-activators. Indeed, some muscle-specific lncRNAs that control muscle gene expression have been reported, including steroid receptor RNA activator^21^, muscle-specific linc-MD1 (ref. 22), two enhancer RNAs transcribed from the upstream regulatory region of MyoD^23^ and Yam-1 (ref. 24). Recently, a lncRNA Dum was reported to regulate *Dppa2* expression by interacting with Dnmts during myogenic differentiation and muscle regeneration^25^.

Here we describe the identification and characterization of a lncRNA Linc-RAM (Linc-RNA Activator of Myogenesis), which is specifically expressed in skeletal muscle tissue and functionally promotes myogenic differentiation. Significantly, *Linc-RAM* KO mice have reduced the number of the myofibers and delayed muscle regeneration. Mechanistically, we reveal that Linc-RAM acts as a regulatory lncRNA directly interacting with MyoD to facilitate assembly of the MyoD–Baf60c–Brg1 complex.

## Results

### Linc-RAM is a muscle expressed and MyoD-regulated lncRNA

To identify MyoD-regulated lncRNAs involving in myogenic differentiation, we analysed public database of RNA-Seq^26^ and MyoD chromatin immunoprecipitation (ChIP)-Seq data^27^ during C2C12 cell differentiation. Forty-five differentially expressed lncRNAs with MyoD-binding peaks within their promoter regions were identified by the integrated analysis (Supplementary Fig. 1). Compared with the similar analyses published from other three independent groups^28^,^29^,^30^, out of 45 lncRNAs, 2 lncRNAs (1600020E01Rik and 2310015B20Rik) were reported as enriched lncRNAs in myotubes^29^ and 1 lncRNA 2310043L19Rik was described in the previous work^30^. We further identified muscle-specifically expressed lncRNAs by examining expression patterns of the identified 45 lncRNA genes in various tissues of mouse. One lncRNA NR_038041 (2310015B20Rik), named as Linc-RAM in the study, was specifically expressed in mouse skeletal muscle cells (Supplementary Fig. 2). By using various approaches, we also demonstrated that *Linc-RAM* was transcriptionally regulated by MyoD both *in vitro* and *in vivo* (Supplementary Fig. 3). Syntenic region analysis suggests human version of *Linc-RAM* is likely Linc-00948 that has been annotated as a lncRNA in human genome (Supplementary Fig. 4). Intriguingly, Linc-RAM happens to be the putative lncRNA encoding a recently identified micropeptide myoregulin (MRLN)^31^, which mediates muscle performance by regulating Ca2^+^handling through inhibiting the pump activity of SERCA (Sarco endoplasmic reticulum calcium adenosine triphosphatase)^31^.

### Linc-RAM promotes myogenic differentiation

**Figure 1:**
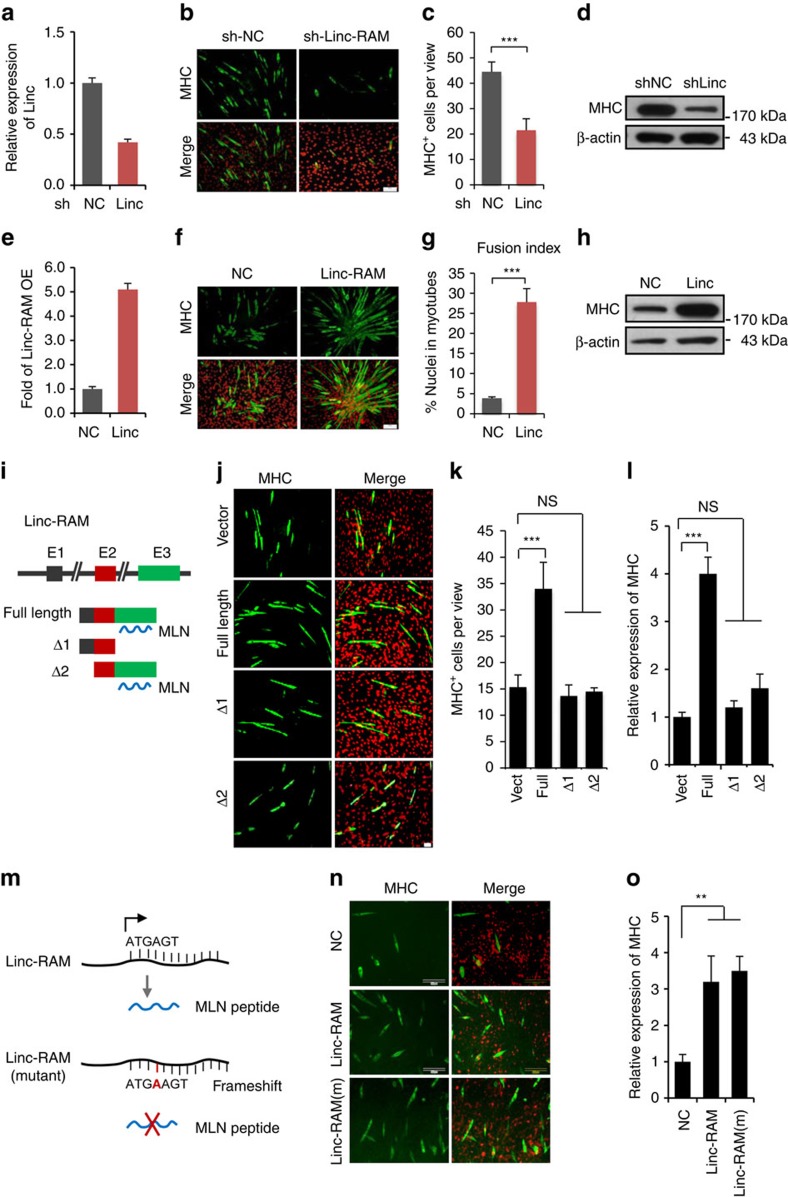
Linc-RAM enhances myogenic differentiation in a MRLN-independent manner. (**a**) Linc-RAM was knocked down in C2C12 cells. Knockdown efficiency was examined by RT–qPCR. (**b**) The differentiation of Linc-RAM knockdown C2C12 cells was assayed by staining for MHC at 48 h in differentiation medium (DM). Scale bars, 50 μm. (**c**) MHC^+^ cells in **b** were counted. (**d**) MHC expression in (**b**) was detected by western blotting. β-actin served as a loading control. (**e**) Linc-RAM was overexpressed in C2C12 cells using a lentivirus system. The degree of Linc-RAM overexpression (fold increase) was determined by RT–qPCR. (**f**) The differentiation of C2C12 cells stably overexpressing Linc-RAM was examined by MHC staining at 48 h in DM. Scale bar, 50 μm. (**g**) Fusion index in **f** were calculated. (**h**) MHC expression in **f** was detected by western blotting. β-actin served as a loading control. (**i**) Schematic illustration of the plasmids for full-length Linc-RAM and two truncation mutants, Δ1 and Δ2; Δ1 contains exons 1 and 2, whereas Δ2 covers exons 2 and 3. MRLN peptide is indicated as blue line. (**j**) Differentiation of C2C12 cells transfected with the full length and truncated Δ1 and Δ2 was examined by staining for MHC after culturing in DM for 36 h. Scale bars, 50 μm. (**k**) MHC+ cells in **j** were counted and presented as positive cells per view. (**l**) MHC mRNA expression in **j** was detected by RT–qPCR. (**m**) Schematic illustration of the plasmids with WT Linc-RAM containing MRLN ORF and mutant Linc-RAM harbouring a frameshift for MRLN ORF. (**n**) Differentiation of C2C12 cells transfected with WT and mutant Linc-RAM was examined by staining for MHC after culturing in DM for 36 h. Scale bars, 100 μm. (**o**) MHC mRNA expression in **n** was detected by RT–qPCR. All images in the figure are representatives of three independent experiments. Values are means±s.e.m. of three independent experiments. The statistical significance of the difference between two means was calculated with the *t*-test. **P<0.01, ***P<0.001. NS stands for statistically non-significant.

Given the fact that Linc-RAM was specifically expressed in skeletal muscle cells and its expression was regulated by MyoD, it was conceivable that Linc-RAM plays a regulatory role in regulating myogenesis. Thus, we first examined the effect of Linc-RAM depletion on myogenic differentiation in C2C12 cells stably expressed two independent of short hairpin RNAs (shRNA) targeting Linc-RAM, respectively (Fig. 1a; Supplementary Fig. 5). Linc-RAM knockdown in differentiating C2C12 cells resulted in a marked decrease of myoblast differentiation into myotubes, as evidenced by a reduced number of myosin heavy chain-positive (MHC^+^) cells (Fig. 1b,c; Supplementary Fig. 5) and lower levels of MHC protein (Fig. 1d), as compared with negative control (NC) cells harbouring a non-targeting shRNA. Conversely, transiently overexpressed full-length Linc-RAM significantly enhanced the myogenic differentiation, by increasing the expression of MyoG and the number of the MyoG^+^ cells (Supplementary Fig. 6). To further support this observation, we stably overexpressed full-length Linc-RAM in C2C12 cells (Fig. 1e) and examined its ability to promote myogenic differentiation by immunostaining with an antibody against MHC. As shown in Fig. 1f and Supplementary Fig. 7, interestingly, we observed the significantly enhanced differentiation and a ‘radial' pattern of the differentiated myotubes from the cells overexpressing Linc-RAM. Consistently, stably overexpressed Linc-RAM significantly enhanced myogenic differentiation, as shown by an increased fusion index Fig. 1g and level of MHC protein (Fig. 1h). To clarify that the pro-myogenic effect is mediated by Linc-RAM ncRNA rather than by its encoded micropeptide MRLN, the truncated mutants of Linc-RAM without (delta 1) or with (delta 2) MRLN open reading frame (ORF) were overexpressed in differentiating C2C12 cells (Fig. 1i) and none of the mutants was able to promote myogenic differentiation (Fig. 1j–l), suggesting that full-length Linc-RAM is required for myogenic differentiation in a MRLN-independent manner. To further confirm this, we overexpressed a frameshift mutant of full-length Linc-RAM in MRLN ORF, in which the MRLN was unable to be translated in the cells (Fig. 1m). Again, we found that the mutated Linc-RAM unable to encode for MRLN promoted myogenic differentiation with the similar efficiency as wild-type (WT) Linc-RAM (Fig. 1n,o). Collectively, our multiple lines of experimental data revealed that functional role of Linc-RAM in promoting myogenic differentiation was MRLN independent.

### *Linc-RAM* KO mice display delayed muscle regeneration

**Figure 2:**
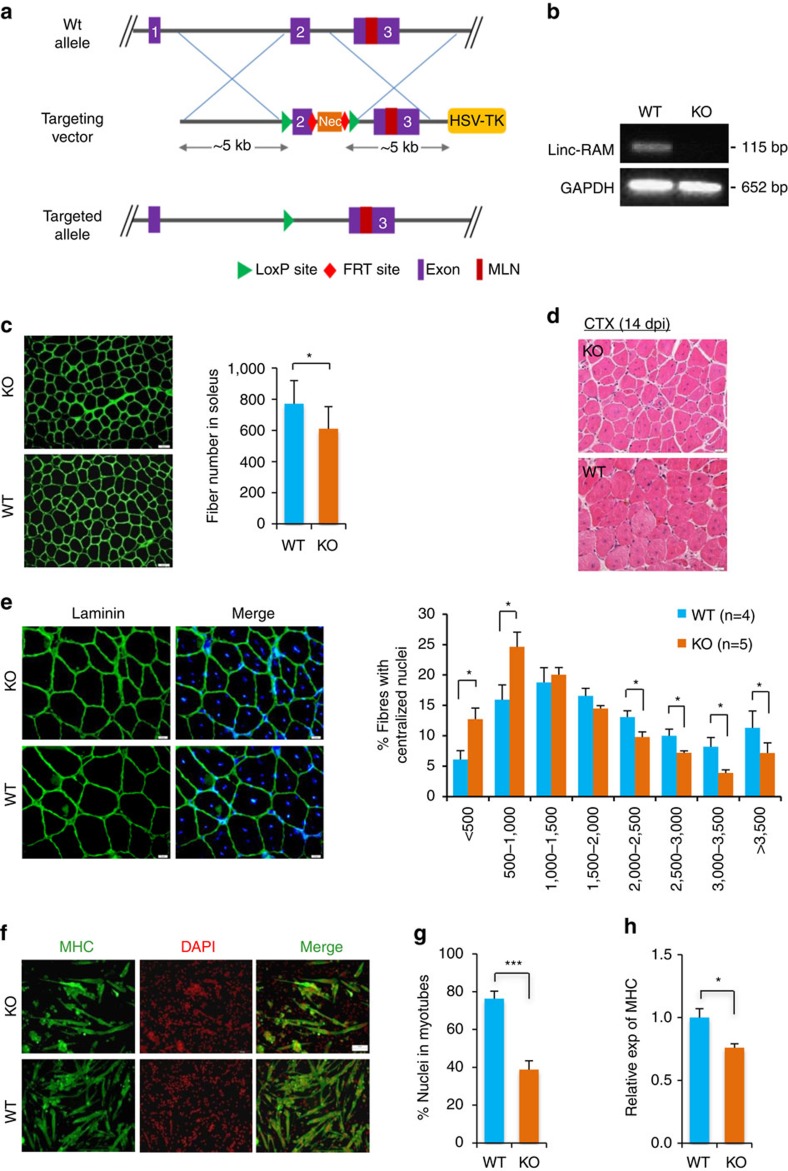
Linc-RAM knockout mice exhibits delayed muscle regeneration. (**a**) Strategy for generation of *Linc-RAM* knockout mice. LoxP sequences were inserted in the flanking of exon 2 of the *Linc-RAM* gene. (**b**) The exon 2 deletion in knockout muscle was confirmed by RT–PCR. (**c**) The numbers of myofibers in soleus muscle of wild-type (WT; *n*=7) and knockout (KO) mice (*n*=9) were calculated based on laminin staining (left). Scale bars, 50 μm. (**d**) Representative hematoxylin and eosin (H&E)-stained sections of TA muscle 14 days post injury (14 dpi) induced by CTX injection. Scale bars, 50 μm. (**e**) Cross-sectional area of regenerated myofibers with centralized nuclei, calculated from the laminin-stained sections (left). Scale bars, 20 μm. (**f**) The differentiation of primary myoblasts isolated from 3-week-old Linc-RAM KO and WT littermates were analysed by inducing differentiation in DM for 36 h and staining with MHC. The presented images are representatives of three independent experiments. Scale bars, 50 μm. (**g**) Fusion index in **f** were calculated. (**h**) The expression of MHC in **f** was detected by RT–qPCR. GAPDH was the internal control. Values are means±s.e.m. The statistical significance of the difference between two means was calculated with the *t*-test, *P<0.05, ***P<0.001.

To strengthen the above *in vitro* findings, we then investigated *in vivo* functional role of Linc-RAM in regulating muscle development and regeneration by generating *Linc-RAM* KO mice. The strategy used for generating *Linc-RAM* KO mice was different from the MRLN KO mice reported by Anderson *et al*.^31^ Only the exon 2 was deleted in our *Linc-RAM* KO mice, while the exon 1 and 3 were still present (Fig. 2a,b), leading to the generation of an exon 1–3 fusion transcript that still contains the intact MRLN ORF (Supplementary Fig. 8a). No overt different in body weight, the muscle mass and myofibers size were observed in the *Linc-RAM* KO mice compared with their WT littermates (Supplementary Fig. 8b–d); however, the number of the myofibers were significantly reduced in *Linc-RAM* KO mice than in WT littermate controls (Fig. 2c). We next investigated how Linc-RAM regulates satellite cell function during muscle regeneration induced by injecting cardiotoxin (CTX) into tibialis anterior (TA) muscle of *Linc-RAM* KO mice and WT mice. During regeneration, *Linc-RAM* expression markedly increased 3 days after injury (Supplementary Fig. 9), suggesting that Linc-RAM regulates satellite cell differentiation during regeneration of damaged muscle in mice. In support of this notion, we found that at 14 days after injection, regenerating myofibers, characterized by centralized nuclei, were significantly smaller in *Linc-RAM* KO mice than in WT littermates control (Fig. 2d,e). Next, we directly evaluated the influence of Linc-RAM on satellite cell differentiation by using freshly isolated satellite cells from hind limb skeletal muscle of *Linc-RAM* KO and WT littermates. The isolated satellite cells were cultured in differentiation medium for 36 h and immunostained with antibody against MHC (Fig. 2f). Consistent with the functional role of Linc-RAM in enhancing C2C12 myogenic cell differentiation (Fig. 1), myogenic differentiation of the isolated satellite cells from the *Linc-RAM* KO mice was significantly delayed, as shown by a decreased fusion index (Fig. 2g) and reduced levels of MHC messenger RNA (mRNA; Fig. 2h). Together, results of both *vitro* and *in vivo* functional assays convincingly reveal the novel role of Linc-RAM in promoting myogenic differentiation during muscle development and regeneration.

### Nuclear Linc-RAM directly interacts with MyoD in muscle cell

**Figure 3:**
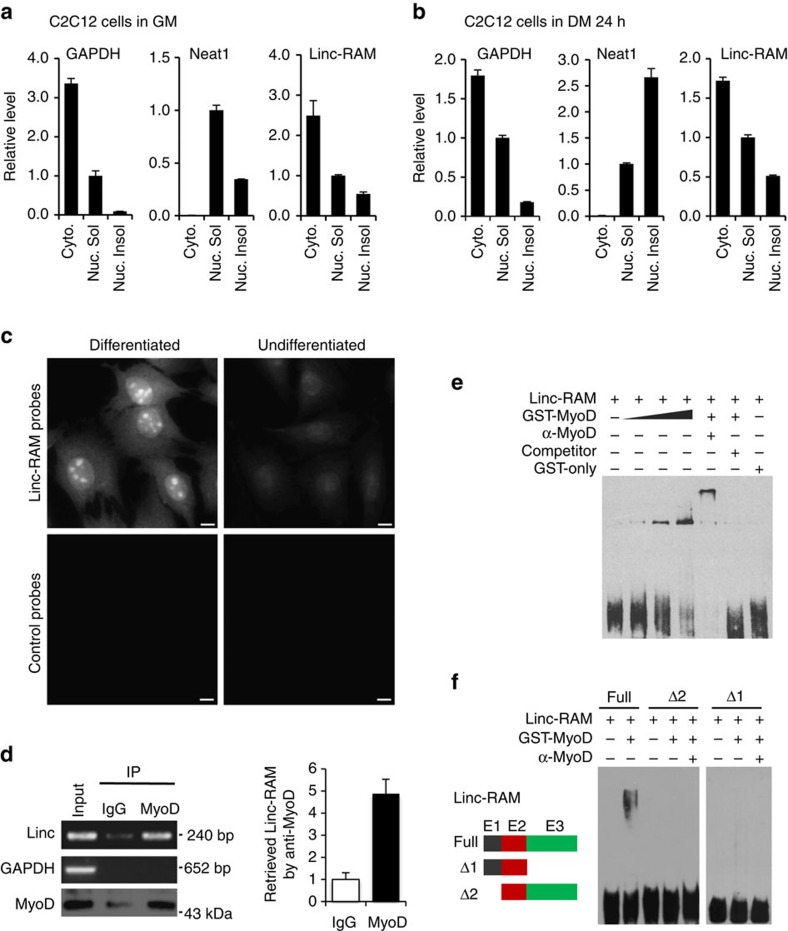
Nuclear Linc-RAM physically interacts with MyoD in the muscle cells. (**a**,**b**) Linc-RAM in cytoplasmic (Cyto), nuclear-soluble (Nuc.Sol) and nuclear-insoluble (Nuc.Insol) fractions of proliferating (**a**) and differentiating (**b**) C2C12 cells was determined by qRT–PCR. Neat1 (nuclear paraspeckle assembly transcript 1) were used as markers for the nuclear fraction; GAPDH was used as markers for the cytoplasmic fraction. The data are representatives of three independent experiments. (**c**) Subcellular localization of Linc-RAM in differentiated and undifferentiated C2C12 cells (DM 2 days) was examined by RNA FISH using a pool of singly-Cal610-labeld ODN probes against the Linc-RAM (Linc-RAM Probes). A pool of singly-Cal610-labeld ODN probes against the EGFP-coding sequence served as nonsense control (control probes). The nuclei were stained with DAPI. The images are representatives of three independent experiments. Scale bar, 20 μm. (**d**) RNA immunoprecipitation (RIP) was used to examine the physical interaction of Linc-RAM with MyoD. Muscle homogenates were immunoprecipitated using anti-MyoD antibodies, and Linc-RAM in immunoprecipitates were detected by semi-qRT–PCR (left) and qRT–PCR (right). GAPDH served as a negative control. MyoD in above immunoprecipitates were detected by western blotting. (**e**) The direct interaction between MyoD and Linc-RAM was examined by electrophoresis mobility shift assay (EMSA) with purified GST–MyoD fusion protein. The presented blot is a representative of three independent experiments. (**f**) The interaction between MyoD and different truncated form of Linc-RAM were examined by EMSA. The presented blot is a representative of three independent experiments.

Functional independence of Linc-RAM in enhancing myogenic differentiation on MRLN supports the notion that Linc-RAM functions as a regulatory RNA in promoting myogenic cells differentiation. To confirm this, we first examined subcellular localization of Linc-RAM and found that the Linc-RAM transcript is present in both nuclei and cytoplasm of myoblasts (Fig. 3a) and myotubes (Fig. 3b), which was also supported by fluorescence *in situ* hybridization (FISH) analyses (Fig. 3c). Collectively, the nuclear localization of Linc-RAM and its MRLN-independent function indicated that Linc-RAM acts as a regulatory lncRNA involved in transcriptional control of muscle genes expression during skeletal muscle development.

Considering that Linc-RNAs can regulate gene expression by interacting with a specific transcriptional factor or a component of chromatin-modifying complexes^3^,^32^ and the nuclear localization of Linc-RAM in the muscle cells, we next tested the possibility that Linc-RAM functions in muscle cells by physically interacting with MyoD in nucleus. We performed RNA immunoprecipitation assays with the nuclear fraction of muscle cells using affinity-purified anti-MyoD antibody and assayed the samples by quantitative PCR with reverse transcription (RT–qPCR) using primers specific for the Linc-RAM transcript. The Linc-RAM transcript was pulled down only by an anti-MyoD antibody and not by an anti-IgG control antibody (Fig. 3d), indicating that Linc-RAM physically associates with MyoD in muscle cells. The glyceraldehyde-3-dehydrogenase (*GAPDH*) transcript, used as a NC, was not detected in the immunoprecipitated samples by RT–PCR (Fig. 3d), confirming the specificity of the anti-MyoD antibody. Next, we performed electron mobility shift assay with GST–MyoD fusion protein to further assess direct interaction between Linc-RAM and MyoD. We found that Linc-RAM directly interacted with MyoD (Fig. 3e) and their specific interaction was evidenced by showing the MyoD antibody mediated super shift (Fig. 3e) and abolished binding with the cold competitor probes (Fig. 3e). To further identify the Linc-RAM-binding domain required for its interaction with MyoD, we generated the different truncated mutants of Linc-RAM (Fig. 3f) and found that all the mutants were unable to physically bind with MyoD (Fig. 3f), indicating that the full length of Linc-RAM is essentially required for its physical interaction with MyoD. Consistent with the results that none of the Linc-RAM mutants was able to promote myogenic differentiation (Fig. 1i–l), our data support the notion that physical interaction of the full-length Linc-RAM with MyoD is required for its function to promote myogenic differentiation. Furthermore, we found that Linc-RAM did not bind MyoG protein (Supplementary Fig. 10), supporting functional role of Linc-RAM in regulating myogenic differentiation by specifically interacting with MyoD. Collectively, our results from both physical binding and functional assays not only provides convincing data to uncover Linc-RAM acting as a regulatory lncRNA for promoting myogenic differentiation in a MRLN-independent manner, but also give a mechanistic explanation for why the truncated mutants cannot promote myogenic differentiation.

### Linc-RAM enhances transcriptional activity of MyoD

**Figure 4:**
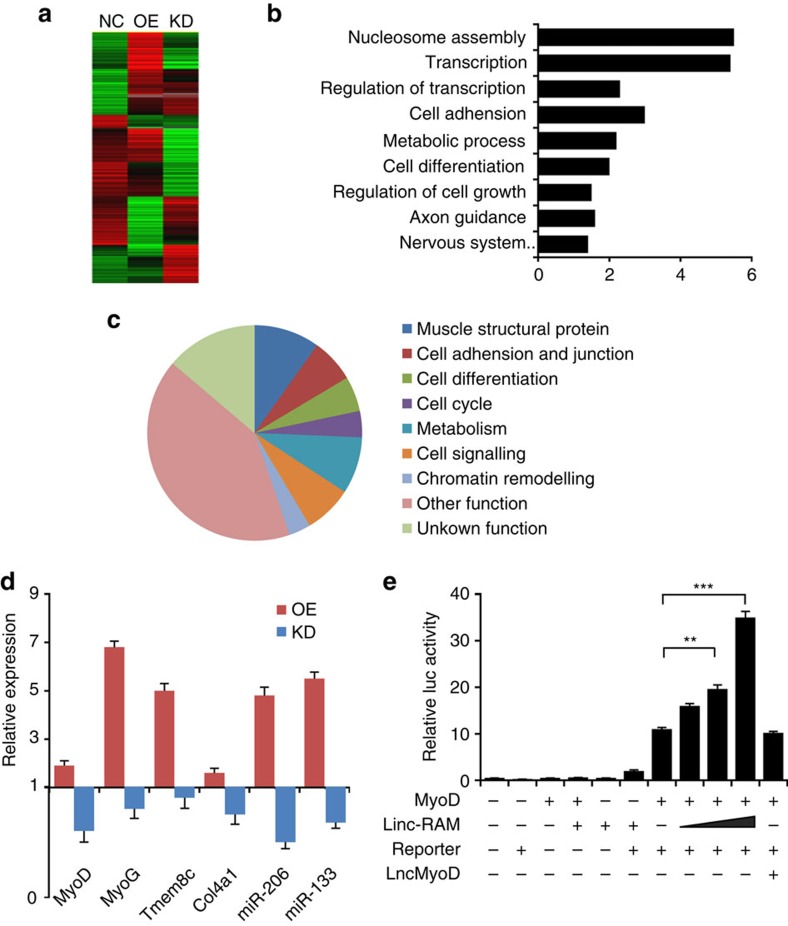
Linc-RAM acts as a regulatory lncRNA enhancer of MyoD in regulating expression of myogenic genes. (**a**) RNA sequencing analyses were applied to stable Linc-RAM-overexpressing (OE) and Linc-RAM knockdown (KD) C2C12 cell lines; cells stably infected with vector alone (NC) served as controls. The heatmap shows hierarchical clusters for 852 differentially expressed genes, which were designated Linc–RAM-affected/targeted genes. Red, upregulated; green downregulated (cutoff, ≥1.5-fold change; *P*≤0.005). (**b**) Enriched GO terms for Linc–RAM-affected genes. The *y* axis shows GO terms and the *x* axis shows statistical significance (negative logarithm of *P* value). (**c**) MyoD ChIP-seq data were applied to analyse whether Linc-RAM-affected genes were also regulated by MyoD. The pie chart demonstrates GO term classifications for 151 Linc-RAM-affected genes with at least one MyoD-binding peak in the promoter region. (**d**) Differentially expressed genes were validated by RT–qPCR. (**e**) Luciferase reporter gene assay to measure *MyoG* promoter activity in fibroblasts cotransfected with MyoD and different amounts of Linc-RAM. LncMyoD, a non-related long non-coding RNA, served as negative control. The data are presented as mean±s.e.m. from three independent experiments. The statistical significance was calculated with the *t*-test, **P<0.01, ***P<0.001.

The directly physical interaction between Linc-RAM and MyoD in the muscle cells suggests that Linc-RAM might act in concert with MyoD to regulate transcription of a common set of myogenic genes. Furthermore, our ChIRP (Chromatin Isolation by RNA Purification) analysis indicated that Linc-RAM is a chromatin-associated linc-RNA, as evidenced by identifying Linc-RAM genomic-binding sites in myogenin gene promoter from the recovered chromatin by quantitative PCR in muscle cells (Supplementary Fig. 11). We, therefore, investigated the global effect of Linc-RAM on gene expression by RNA-Seq analysis during myogenic differentiation using RNAs isolated from differentiating C2C12 myoblasts, in which Linc-RAM was either stably overexpressed or knocked down. First, we found that 264 genes were upregulated and 235 genes were downregulated (≥2 fold difference in expression) in Linc-RAM-overexpressing C2C12 cells compared with control cells. In Linc-RAM knockdown cells, 305 upregulated and 237 downregulated genes were identified (Fig. 4a; Supplementary Data set 1). A gene set enrichment analysis of differentially expressed genes revealed that Linc-RAM-regulated genes were highly enriched for the terms nucleosome assembly and transcriptional regulation of myogenic gene expression (Fig. 4b). These results indicate that Linc-RAM exerts a global effect on the expression of genes involved in myogenic differentiation.

Interestingly, when overlapping the above list of differentially expressed genes with the MyoD ChIP-seq data set, we found 151 of these 882 genes exhibited MyoD-binding peaks in their promoter regions (Supplementary Data set 2). Significantly, this gene set showed highly enrichment for muscle cell proliferation, differentiation and muscle structural proteins (Fig. 4c; Supplementary Table 1). Some coregulated myogenic genes, including those encoding *MyoD*, *MyoG*, *Tmem8c* (transmembrane protein 8C), *Col4a1* (collagen, type IV, alpha 1), miR-206 and miR-133 were confirmed by real-time quantitative RT–PCR (Fig. 4d). These analyses provide molecular evidence for physical interaction between Linc-RAM and MyoD in controlling transcription of a common set of genes required for myogenic differentiation,

To further confirm that Linc-RAM acts in concert with MyoD to synergistically regulate transcription of myogenic genes during myogenic differentiation, we used luciferase reporter gene system driven by the *MyoG* proximal promoter as shown in Supplementary Fig. 12. Luciferase reporter activity was assayed in C3H-10T1/2 fibroblasts transiently transfected with MyoD alone or with Linc-RAM in the presence of the reporter construct. As previously reported, forced expression of MyoD alone in fibroblasts activated the luciferase reporter gene (Fig. 4e). Notably, co-transfected Linc-RAM induced a significant, dose-dependent enhancement of MyoD-mediated luciferase activity in fibroblasts (Fig. 4e). Recently, another MyoD-regulated Linc-RNA named as LncMyoD was reported to regulate myogenic differentiation with a mechanism by which LncMyoD directly binds to IGF2-mRNA-binding protein 2 and negatively regulates IGF2-mRNA-binding protein 2-mediated translation of proliferation genes such as N-Ras and c-Myc^29^. By using the LncMyoD as a NC as shown in Fig. 4e, we concluded that Linc-RAM acts as a specific RNA enhancer of MyoD in mediating transcription of the *MyoG* gene.

### Linc-RAM facilitates formation of MyoD–Baf60c–Brg1 complex

**Figure 5:**
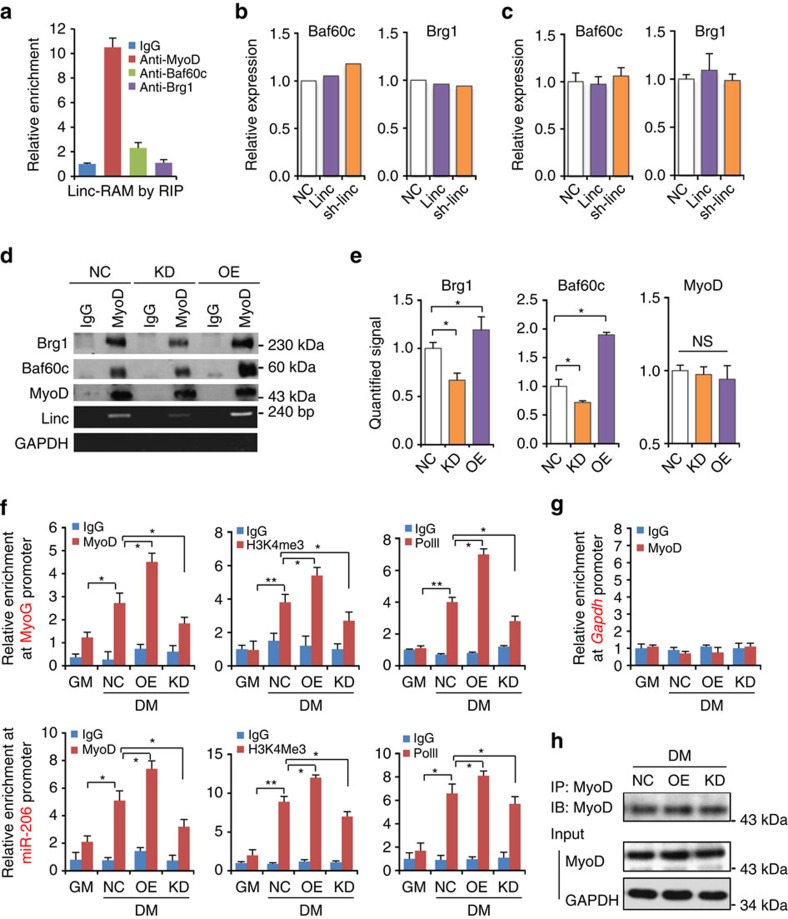
Linc-RAM facilitates formation of the MyoD–Baf60c–Brg1 complex by interacting with MyoD. (**a**) Interaction between Linc-RAM and MyoD, Baf60c and Brg1 determined by RNA immunoprecipitation (RIP). C2C12 cell lysates were immunoprecipitated using anti-MyoD, anti-Baf60c or anti-Brg1 antibodies, and Linc-RAM in immunoprecipitates was detected by RT–qPCR. IgG antibodies served as a control. (**b**) Expression of Baf60c and Brg1 in C2C12 cells with overexpression or knockdown (KD) of Linc-RAM analysed by RNA-Seq. (**c**) Expression of Baf60c and Brg1 in C2C12 cells with overexpression or KD of Linc-RAM determined by RT–qPCR. (**d**) Co-immunoprecipitation of Linc-RAM and the components in the MyoD/Baf60c/Brg1 complex determined by RIP analysis. Linc-RAM-overexpressing (OE) and Linc-RAM KD C2C12 cell lysates were immunoprecipitated using MyoD antibodies; Baf60c, Brg1 and MyoD in immunoprecipitates were detected by western blotting, and Linc-RAM was detected by RT–PCR. GAPDH served as a negative control. (**e**) Quantification of the immunoprecipated products in **d**. (**f**) ChIP assays were performed using chromatin from stable Linc-RAM-OE and Linc-RAM KD C2C12 cell lines and negative control (NC) cells cultured in growth medium (GM) or differentiation medium (DM). Chromatin was immunoprecipitated using antibodies against MyoD, H3K4me3, and RNA Pol II. The immunoprecipitated DNA was amplified using primers specific for *MyoG* and *miR-206* gene promoters. (**g**) Gapdh gene promoter were amplified using the same samples presented in **f**. (**h**) The resulted immunoprecipitates in **f** were applied for detection of MyoD by western blotting. The data are presented as mean±s.e.m. from three independent experiments. The statistical significance was calculated with the *t*-test, *P<0.05, **P<0.01.

It has recently been reported that MyoD physically associates with the SWI/SNF subunit on regulatory elements of MyoD-target genes in myogenic precursor cells, thereby facilitating incorporation of MyoD–BAF60c into a Brg1-based SWI/SNF complex involved in myogenic differentiation. The MyoD–BAF60c–Brg1 complex, in turn, remodels the chromatin of MyoD-target genes, enabling their subsequent transcription^20^. To further explore the mechanistic insights of how Linc-RAM regulates MyoD transcriptional activity, we tested whether Linc-RAM might be involved in formation of the MyoD–BAF60c–Brg1 complex. We first examined whether Linc-RAM interacts with Baf60 or Brg1 by RNA immunoprecipitation assays using antibodies against Baf60c and Brg1. As shown in Fig. 5a, Linc-RAM was not pulled down with either Baf60c or Brg1 proteins, indicating that Linc-RAM did not directly associate with Baf60c or Brg1. We then asked whether Linc-RAM regulates transcription of *Baf60c* and *Brg1* by using the RNA-Seq data described in Fig. 4. The analysis revealed that neither overexpression nor knockdown of Linc-RAM in differentiating C2C12 cells altered the levels of *Baf60c* or *Brg1* mRNA compared with control cells (Fig. 5b), which was further validated by real-time RT–PCR assay (Fig. 5c). Together, our results demonstrate that Linc-RAM was not associated with Baf60c and Brg1, nor did its association with MyoD regulate transcription of *Baf60c* and *Brg1* in the differentiating cells.

The observations shown in Fig. 5a–c prompted us to propose that the physical association of Linc-RAM with MyoD might affect the interaction between MyoD and Baf60 in muscle cells. For this purpose, we immunoprecipitated proteins from differentiating C2C12 cells stably overexpressing Linc-RAM or shRNA targeting Linc-RAM with an anti-MyoD antibody, and then immunoblotted the immunoprecipitated proteins using antibodies against Baf60c and Brg1. Remarkably, the amount of Baf60c and Brg1 proteins detected in immunoprecipitated MyoD–Baf60c–Brg1 complexes was increased in cells overexpressing Linc-RAM (Fig. 5d,e). Conversely, Linc-RAM knockdown resulted in a decrease in Baf60c and Brg1 protein levels in the MyoD–Baf60c–Brg1 complex (Fig. 5d,e), suggesting that Linc-RAM facilitated MyoD–Baf60c–Brg1 complex formation through interactions with MyoD.

Next, we further evaluated this mechanism of Linc-RAM action by ChIP assay using chromatin isolated from differentiating C2C12 cells stably overexpressing Linc-RAM or shRNA targeting Linc-RAM. MyoD binding to the endogenous *MyoG* promoter was assessed by immunoprecipitating isolated chromatin with an antibody against MyoD and then analysing bound DNA fragments by RT–qPCR using primers that amplify regulatory regions of the *MyoG* gene. As shown in Fig. 5f, MyoD was enriched in the *MyoG* promoter of differentiating C2C12 cells overexpressing Linc-RAM compared with control cells. In contrast, enrichment of MyoD at the *MyoG* promoter was attenuated in Linc-RAM knockdown C2C12 cells. We also analysed the same immunoprecipitated DNA for enrichment of the *miR-206* promoter using an anti-MyoD antibody and found the results were consistent with those obtained for the *MyoG* promoter (Fig. 5f). Moreover, transcriptional initiation of both *MyoG* and *miR-206* genes was significantly enhanced in Linc-RAM-overexpressing C2C12 cells relative to Linc-RAM knockdown and control cells, as evidenced by ChIP data obtained using antibodies against H3K4Me3 and RNA polymerase II (Fig. 5f). No any enrichment of MyoD binding at the *GAPDH* gene promoters (Fig. 5g) and equal efficacy of immunoprecipitation with anti-MyoD (Fig. 5h). Taken together, our findings uncover that Linc-RAM functionally acts as a regulatory lncRNA enhancer of MyoD in the transcriptional regulation of genes required for myogenic differentiation by facilitating MyoD–Baf60c–Brg1 complex formation through interactions with MyoD.

## Discussion

**Figure 6:**
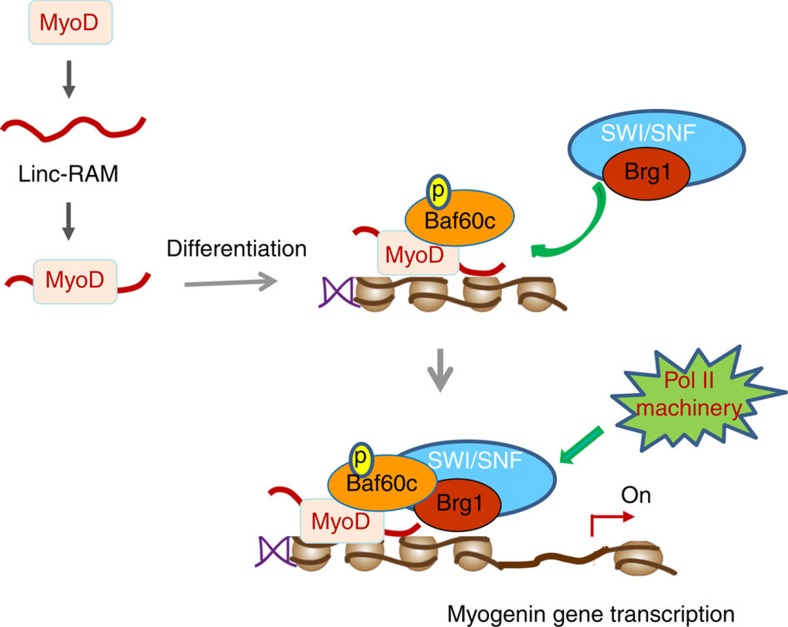
Hypothetical working model of Linc-RAM functions. Linc-RAM is transcriptionally upregulated by MyoD and directly binds to MyoD in muscle cells. In differentiating cells, Linc-RAM facilitates the recruitment of the SWI/SNF core to myogenic gene promoters through interaction with MyoD, thereby promoting chromatin remodelling and assembly of the transcriptome for transcriptional initiation of myogenic differentiation genes.

Recent studies using a KO mouse approach have highlighted the importance of lncRNAs in regulating the cell differentiation and development^2^. Even though a numbers of the lncRNAs have been identified in various biological systems, for most of them there are *in vivo* physiological function and key mechanistic aspects that remain unexplored, including selective expression in specific cell types and downstream targets, especially *in vivo* functional evaluation of their significance during development. Myogenic cell differentiation is an excellent and well-characterized system for investigating the genetic and epigenetic regulation of gene expression. Given the functional significance of both MyoD, as a master regulator of myogenic gene transcription, and lncRNAs, as epigenetic regulators of gene transcription during the cell differentiation and development, we asked whether lncRNAs act in concert with MyoD to regulate myogenesis during development. Here we uncover a novel role of the muscle-specifically expressed lncRNA Linc-RAM in regulating the myogenic differentiation both *in vitro* and *in vivo*. Linc-RAM is expressed in satellite cells of mice and Linc-RAM KO mice exhibit delayed muscle regeneration due to differentiation defect of satellite cells. We then propose a model (Fig. 6) to mechanistically reveal the role of Linc-RAM in regulating myogenic differentiation by directly binding MyoD, which promotes the assembly of the epigenetic regulatory complex MyoD–Baf60c–Brg1 on the regulatory elements of target genes.

Recently, emerging reports are beginning to reveal dual functional nature of lncRNAs but examples of such lncRNAs with solid experimental evidence are still lacking. Anderson *et al*. recently demonstrated that the micropeptide MRLN is encoded by a putative Linc-RNA that happens to be Linc-RAM identified in this study. MRLN encoded by Linc-RAM plays a role in regulating Ca2^+^handling by inhibiting the pump activity of SERCA in the muscle cells^31^. In addition to its coding function, our current data from molecular biology, cellular biology, mouse genetics and high-throughput sequencing approaches support the notion that Linc-RAM also acts as a regulatory lncRNA functionally playing a novel role in promoting myogenic differentiation in a MRLN-independent manner. Therefore, distinct from the micropeptide-mediated function, we have uncovered the chromatin regulatory function of this lncRNA Linc-RAM. Conceptually, our findings complemented by Anderson's study uncovered Linc-RAM as one of the very few lncRNAs proven to be functional as both coding and non-coding RNA, which underscores a very novel idea that a given lncRNA may in fact hide-coding potential.

A few lncRNAs with enhancer functions in the transcriptional regulation of coding genes have been reported, including Evf-2 (ref. 33), heat-shock RNA-1 (ref. 34), steroid receptor RNA activator^35^ and enhancer RNAs^36^. Interestingly, MyoD transcriptional activity was significantly increased by Linc-RAM in muscle cells, indicating that Linc-RAM enhances the function of MyoD transcriptional activity. Furthermore, RNA-Seq analysis of genes globally regulated by Linc-RAM identified a subset of Linc-RAM-affected genes known to be also regulated by MyoD. In light of a functional requirement of both Linc-RAM and MyoD in myogenic differentiation and common set of myogenic genes regulated by Linc-RAM and MyoD, our data support the notion that Linc-RAM acts as a lncRNA enhancer of MyoD and synergistically regulates the transcription of myogenic genes in concert with MyoD to mediate myogenic differentiation. Collectively, our findings provide a molecular explanation for the Linc-RAM-mediated enhancement of MyoD function in C2C12 cell differentiation and muscle regeneration in mice.

The transcriptional activity of MyoD in driving myogenic gene transcription is primarily regulated by its interaction with cofactors such as chromatin modifiers and remodelers^20^,^37^. BAF60c (SMARCD3), a structural component of the SWI/SNF chromatin-remodelling complex, plays a regulatory role in the induction of myogenic gene transcription during skeletal muscle development^17^,^37^,^38^,^39^,^40^. Forcales *et al*. have recently reported that MyoD physically interacts with Baf60c and showed that this interaction is required for the recruitment of the Brg1-based SWI/SNF remodelling complex to the promoters of myogenic genes^17^,^20^. However, the molecular details underlying MyoD and Baf60c interactions are unknown. In this report, we provide experimental evidence showing that Linc-RAM physically associates with MyoD and binding of Linc-RAM to MyoD is required for formation of the MyoD–Baf60c–Brg1 complex on promoters of myogenic genes. Our findings uncover a novel molecular mechanism, in which Linc-RAM is an essential lncRNA component required for formation of MyoD–Baf60c–Brg1 complex on the promoters of the muscle-specifically expressed genes during myogenesis. Moreover, our findings conceivably suggest that Linc-RAM, as a new determinant of myogenic differentiation, might functionally acts as an lncRNA cofactor in guarding the specificity of MyoD transcriptional activity during development.

## Methods

### Pipelines for the discovery of MyoD-regulated Linc-RNAs

Mouse RefSeq genes (mm9) were downloaded from the UCSC website. Only RefSeq RNAs with NR_Accession Numbers were retained for further analysis. The public RNA-seq data was downloaded from Gene Expression Omnibus (GEO) (GSE20846). This data set contains >430 million pair-end 75-bp RNA-seq reads from C2C12 cells representing a differentiation time series that includes 0 h (growth medium), and 60 h, 5 days and 7 days after adding differentiation medium^26^. The candidates with summed RPKM (Reads Per Kilobase of transcript per Million mapped reads) values >1 were carried forward for analysis. An overview of expression profiles across different time points was provided by performing hierarchical cluster analysis using Cluster 3.0 (ref. 41) and visualized with TreeView 1.60 (Michael Eisen, Stanford University. http://rana.lbl.gov/EisenSoftware.htm). RPKM values were adjusted by log transformation, mean centring and normalizing genes before clustering. MyoD-regulated Linc-RNAs were identified using published ChIP-seq data downloaded from the Sequence Read Archive (SRA) database using accession codes SRP001761 and SRA010854. This data set contains 11 subsets corresponding to different muscle cell types. The reads were aligned to the mouse genome (mm9) using Bowtie^42^, which requires a single best placement of each read. All reads with multiple alignments were removed. MyoD-binding sites were found using two subsets: SRX016192 (mouse embryo Myf-5/MyoD-null fibroblasts transduced with pCLBABE-Myod retrovirus) and SRX016194 (mouse embryo Myf-5/MyoD-null fibroblasts transduced with control pCLBABE retrovirus). Binding sites were determined from the aligned reads using SISSR (Site Identification from Short Sequence Reads)^43^ with a *P*<0.05. A given Linc-RAM was considered to be MyoD-regulated if it contained MyoD-binding sites in its upstream 5 kbp to downstream 0.5 kbp region (relative to its start site in RefSeq). For display purposes, binding intensity profiles along the genome were calculated at a resolution of 25 bp for all ChIP-seq data. At each position, the number of uniquely aligned reads oriented toward it within a 100-bp flanking region was counted.

### C2C12 cell culture and differentiation

Mouse C2C12 cells (ATCC, CRL-1772) were cultured in growth medium consisting of Dulbecco's modified Eagle's medium (Gibco) supplemented with 4.5 g l^−1^ glucose, 10% fetal bovine serum, 1% antibiotics at 37 °C in a 5% CO_2_ atmosphere. For differentiation of C2C12 myoblasts into myotubes, cells were transferred to Dulbecco's modified Eagle's medium containing 2% horse serum and 1% penicillin and streptomycin, and then cultured for the indicated number of differentiation days. All cells were grown to ∼80–90% confluence before induction of differentiation.

### Linc-RAM knocking down

BLOCK-iT Lentiviral RNAi Expression System (invitrogen) was used to generate the lentiviral Linc-RAM shRNA. Briefly, the shRNA sequences were cloned to the pENTR/U6 Entry vector. The pLenti6/BLOCK-iTTM expression construct was then generated by recombination with pLenti6/BLOCK-iTTM-DEST vector. The sequences for shRNA against Linc-RAM were shown as follows:

shRNA-1 targeted to 249–269 of the Linc-RAM (GGTACTGATCTCTACTACTTC). shRNA-2 targeted to 372–394 of the Linc-RAM (GCAACCTGACTTTCTTTACTC).

### Overexpression of Linc-RAM

For Linc-RAM overexpression, the Linc-RAM sequences were cloned into pVirus-EGFP vector, which was generated from recombination of pEGFP-N1 and pENTR/U6 Entry vector. Briefly, the fragment from U6 promoter to PolIII terminator in pENTR/U6 Entry vector were removed and replaced with CMV-MCS-EGFP sequence from the pEGFP-N1 plasmid. The engineered plasmid, named as pVirus-EGFP containing AttL1 and AttL2, was used for either transiently overexpression or further recombined with pLenti6/BLOCK-iTTM-DEST vector for lentivirus-mediated overexpression of interest genes. In this study, Linc-RAM was cloned into pVirus with EcoRI/NotI restriction enzyme sites. The resulting plasmid pVirus-Linc-RAM was used for either transient overexpression or lentivirus-mediated Linc-RAM stable overexpression in cells.

### Generation of stable cell lines

The BLOCK-It Lentiviral RNAi Expression System (Invitrogen) was used to establish stable cell lines, as described by the manufacturer. Briefly, shRNA sequences were inserted into the pENTRTM/U6 Entry Construct pU6 plasmid and recombination reactions with pLenti6/BLOCK-iTTM-DEST plasmid were performed to yield the pLenti6/BLOCK-iTTM expression construct. HEK293 cells were then co-transfected with this expression construct and the optimized packaging mix, after which the viral supernatant was collected and added to C2C12 cells. Stably transduced cells were selected by incubating in the presence of blasticidin.

### Immunofluorescence staining

Cells were washed with PBS, fixed by incubating with 4% formaldehyde for 10–15 min and permeabilized with 0.1% Triton X-100. After blocking non-specific binding by incubating with 3% bovine serum albumen (BSA) in PBS for 10 min, cells were incubated with anti-MHC (MF20-c) or anti-MyoG (F5D-c) primary antibody (Developmental Studies Hybridoma Bank (DSHB)) in 3% BSA/PBS (1:200 dilution) for 1–1.5 h, washed five times with PBS, then incubated with fluorescein isothiocyanate-conjugated secondary antibody (Zhong Shan Jin Qiao in China, #ZF-0312, 1:200 dilution), prepared as described for primary antibodies, for 0.5 h. Cells were then washed five times with PBS, incubated with 4′,6-diamidino-2-phenylindole (DAPI) for 3 min, washed twice with PBS and examined by fluorescence microscopy. All immunostaining with MHC were performed in three independent experiments. The data from three independent experiments were included in the Supplementary Fig. 13. A representative from three individual data was included in main text Figures.

### Generation of Linc-RAM KO mice

All animal procedures were approved by the Animal Ethics Committee of Peking Union Medical College (ACUC-A01-2016-003). Linc-RAM KO mice in C57BL/6 background were generated by the Model Animal Research Center of Nanjing University. LoxP sequences were inserted in the flanking of exon 2 of the *Linc-RAM* gene. The exon 2 deletion in KO muscle was validated by RT–PCR. The gender- and age-matched littermates of the Linc-RAM KO and WT mice (male) were used for all phenotypic analysis throughout the study.

### Muscle injury and regeneration

Muscle regeneration was induced by injections of CTX (Sigma). Mice were anaesthetized by intraperitoneal injection of ketamine (10 mg kg^−1^) and xylazine (1 mg kg^−1^). For monitoring muscle regeneration, muscle injury was induced in 8-week-old male mice by injecting CTX (50 μl of 10 μM CTX in PBS) into the mid-belly of the right TA muscle. As an internal control, the left TA muscle of each mouse was injected with PBS (50 μl). TA muscles were harvested 14 days after CTX injection to assess the completion of regeneration and repair.

### Isolation and culture of primary myoblasts

Primary myoblasts were isolated from hind limb skeletal muscle of male Linc-RAM KO and WT littermates at 3 weeks old, minced, and digested in a mixture of type I collagenase and Dispase B (Roche Applied Science). Cells were filtered from debris, centrifuged, and cultured in growth medium (F-10 Ham's medium supplemented with 20% fetal bovine serum, 4 ng ml^−1^ basic fibroblast growth factor and 1% penicillin–streptomycin) on collagen-coated cell culture plates at 37 °C in 5% CO_2._

### RNA-Seq data analysis

Raw-sequencing data were mapped to the mouse genome mm9 assembly using the TopHat^44^ with default parameters. DEGSeq^45^ was used to calculate the read coverage for each gene. Related data were submitted to GEO with the accession number GSE72601. Differentially expressed genes were filtered using a change greater than twofold as a criterion for differential expression. Gene set enrichment analysis was performed by GeneMerge^46^ with the gene association file download from GO (version 03/06/2014). Differentially expressed genes were validated using the iQ5 Multicolor Real-Time PCR Detection System (Bio-Rad). The primer sequences were designed using DNAMAN.

### RT–qPCR analysis

Total RNA was extracted using the TRIzol reagent (Life Technologies) and reverse transcribed using RevertAid reverse transcriptase (Thermo Scientific). qPCR analyses were performed using the iQ5 Multicolor Real-Time PCR Detection System (Bio-Rad). All primers used in the study were presented in Supplementary Table 2.

### Nuclear–cytoplasmic fractionation

Cells were washed twice with ice-cold PBS then lysed in ice-cold PBS/0.1% NP-40 containing a protease inhibitor cocktail (Calbiochem) and ribonucleoside–vanadyl complex (10 mM; New England BioLabs). After a brief centrifugation step, the supernatant was collected as the cytoplasmic fraction, and the remaining pellet, following additional washing, was considered the nuclear fraction. The pellet containing nuclei was extracted with cold nuclear lysis buffer (50 mM Tris-HCl pH 8.0; 500 mM NaCl; 1.5 mM MgCl_2_; 0.5% NP-40; 2 mM vanadyl–ribonucleoside complex). The suspension was centrifuged at 16,360*g* for 20 min. The resulting supernatant is corresponding to the nuclear-soluble fraction and the remaining pellet corresponds to the nuclear-insoluble chromatin-associated fraction.

### Immunoprecipitation and RNA immunoprecipitation

Cells were lysed with cell lysis buffer (Cell Signaling Technology) supplemented with protease inhibitor cocktail (Calbiochem, La Jolla, CA). Protein concentrations in extracts were measured using a bicinchoninic acid assay (Pierce). A volume of extract containing 200 μg protein was immunoprecipitated, subjected to SDS–polyacrylamide gel electrophoresis and transferred onto polyvinylidenedifluoride membranes. For RNA immunoprecipitation assays, RNase Inhibitor (40 U μl^−1^; TaKaRa) and protease inhibitor were added to the cell lysis buffer, and ribonucleoside–vanadyl complex (10 mM; New England BioLabs) was added to the wash buffer. Antibodies specific to Baf60c were a gift from Dr Pier Lorenzo Puri (Forcales, 2012); anti-MyoD (Santa Cruz, SC-760) and anti-Brg1 (Santa Cruz, sc-17796X) were obtained commercially. Horseradish peroxidase (HRP)-conjugated secondary antibodies were from Cell Signaling Technology (Beverly, MA, USA).

### Northern blotting

Total RNA extracted from mouse tissues, including heart, liver, brain, lung, kidney, intestine, spleen and skeletal muscle, at 8 weeks of age were separated by PAGE (7 M urea) on 6% polyacrylamide gels and transferred to a nylon membrane (N+; Amersham). Linc-RAM probes were labelled with α-^32^p-cytidine triphosphate (CTP) using DNA polymerase 1 Large (Klenow) Fragment (Promega, U1100). RNA blots were hybridized in ULTRAhyb (Ambion) at 68 °C overnight, washed twice (5 min) with 2 × saline sodium citrate (SSC)/0.1% SDS wash buffer at 68 °C, followed by stringent washes (2 × 30 min) with 0.1 × SSC/0.1% SDS wash buffer at 68 °C. RNA blots were then exposed to X-ray film at −80 °C. Full scan of Northern blot were presented in Supplementary Fig. 14.

### Combined ChIP–qPCR assay

ChIP analyses were performed on chromatin extracts from Linc-RAM-overexpressing and Linc-RAM knockdown C2C12 cells according to the manufacturer's standard protocol (Millipore, Cat. #17-610) using antibodies against the following proteins: MyoD (Santa Cruz, SC-760), RNA polymerase II (Covance, MMS-126R) and trimethyl-histone H3 (Lys4; Millipore, Cat. #07-473). Fold enrichment was quantified using qRT–PCR.

### Chromatin Isolation by RNA Purification

ChIRP experiment was performed with kit from Millipore (Catalogue No. 17-10495) following procedures in manual instruction. In brief, C2C12 cells grown in differentiation medium for 24 h were trypsinized with 0.25% trypsin and washed with 1 × PBS buffer. The cells (2 × 10^7^) were then crosslinked with 20 ml of 1% glutaraldehyde PBS at room temperature (18–25 °C) for 10 min on an end-to-end rotator. The excess glutaraldehyde was quenched by adding 2 ml of 1.25 M glycine with incubation for additional 5 min. After washed with 20 ml of cold PBS, the crosslinked cells were resuspended in 2 ml of lysis buffer and sonicated with a Bioruptor (Diagenode) in a cold room using the following parameters: H—high setting, pulse interval—30 s ON and 30 s OFF, 10 repeats. After nine cycle sonication, the fragmented chromatin (100–500 bp in length) was split into two parts and hybridized with BiotinTEG-labelled tiling probes against Linc-RAM (Supplementary Table 3) and LacZ control probes, respectively, in hybridization buffer at 37 °C for 4 h. After 4 h hybridization reaction, 100 μl of of PureProteome Streptavidin magnetic beads were added into each reaction and incubated at 37 °C for additional 30 min. The beads were washed four times using prewarmed washing buffer at 37 °C for 5 min. The retrieved beads–RNA–protein–DNA complex was split into two parts: 1/10 for RNA isolation and 9/10 for DNA purification. Finally, the retrieved RNA and DNA were analysed by real-time quantitative PCR and the data were presented as percentage of input RNA and DNA, respectively.

### RNA electrophoresis mobility shift assay right

Biotin-labelled RNA probe was generated by *in vitro* transcription with T7 RNA polymerase (Fermentas) with biotin-UTP (Ambion). DNA templates were digested with DNase I (Promega), then RNA probe were purified by extraction with TRiZol reagent (Ambion). The labelled RNA probe was incubated with appropriate amounts of recombinant proteins in binding buffer (10 mM Tris (pH 7.5), 1 mM EDTA, 0.1 M KCl, 0.1 mM DTT, 5% v/v glycerol and 0.01 mg ml^−1^ BSA) with transfer RNA carrier at room temperature for 30 min. The reactions were then loaded onto 5% native polyacrylamide gel and transferred to Nylon membrane (Amersham). The blot was incubated with HRP-Streptavidin (Invitrogen) and subsequently detected with ECL reagents (Thermo Scientific).

### Luciferase reporter assay

Promoter activity was assessed by transiently transfecting C2C12 cells with a promoter-luciferase reporter plasmid using the FuGene HD transfection reagent (Roche). Twenty-four hours later, the luciferase activity in cell lysates was determined using the Dual-Luciferase Reporter Assay System (Promega) according to the manufacturer's instructions. The plasmid of luciferase reporter driven by a 575 bp basal myogenin promoter (GBBS) was gifted from Dr Zhenguo Wu (Dept of Biochemistry, The Hong Kong University of Science and Technology, Hong Kong).

### Fluorescence *in situ* hybridization

FISH was performed as previously described with modifications^47^. In brief, cells previously cultured in Lab-Tek chambered coverglasses coated with fibronectin were fixed in a 1 × PBS solution containing 4% (wt/vol) paraformaldehyde for 30 min at room temperature, washed with 1 × PBS and permeabilized at 4 °C in 70% (vol/vol) ethanol overnight. On the following day, the cells were washed three times with wash buffer (2 × SSC, 10% (vol/vol) formamide) and then incubated in hybridization buffer (10% (wt/vol) dextran sulfate, 2 × SSC, 10% (vol/vol) formamide) containing a pool of singly-Cal610-labelled ODNs that are complementary to different regions of the Linc-RAM RNA (Biosearch Technologies) or a pool of singly-Cal610-labelled ODNs^48^ that are complementary to different regions of the EGFP-coding sequence (concentration=250 nM; Biosearch Technologies) for 24 h at 37 °C in a humidified chamber. Samples were washed three times with wash buffer followed by 2 × SSC to remove unbound probes, and incubated in 1 × PBS containing DAPI before imaging. Fluorescence imaging were performed on an Olympus IX 83 motorized inverted fluorescence microscope equipped with a × 60 PlanApo N 1.42 numerical aperture objective lens, back-illuminated electron multiplying charge coupled device (EMCCD) camera (Andor), Sutter excitation and emission filter wheels and an MT-20E excitation source (Olympus) controlled by CellSens Dimension software. Images were acquired using the Olympus MT20 filter set for DAPI and a Chroma filter set for CAL Fluor Red 610 (Cal610; ET560/ × 40, ET630/75m, T585lpxr, Chroma). All images were analysed with Fiji^49^. The sequences of RNA probes against Linc-RAM were included in Supplementary Table 3.

### Whole-mount ISH

Whole-mount ISH on mouse embryos was performed as described in the whole-mount ISH protocol for mRNA detection ( http://geisha.arizona.edu/geisha/protocols.jsp). In brief, E11.5 mouse embryos were dissected from the decidua in PBS and fixed in 10 ml of fresh fixative (4% paraformaldehyde in PBS) at 4 °C overnight. The fixed embryos were washed twice and treated with 10 μg ml^−1^ proteinase K in PBS-T for 20 min. The embryos were briefly rinsed with PBS-T and post fixed for 20 min in 4% paraformaldehyde in PBS-T. After rinsed with PBS-T, the embryos were incubated in 1:1 PBS-T/hybridization buffer for 5 min and prehybridized with 1 ml of fresh hybridization buffer for 24 h at 65 °C. Then, the embryos were incubated in 1 ml of prewarmed hybridization buffer and ∼1 μg ml^−1^ digoxin (DIG)-labelled RNA probe for 48 h at 65 °C with gently shaking. The embryos were washed twice for 30 min each with prewarmed (65 °C) hybridization buffer and washed for 10 min at 65 °C with prewarmed 1:1 hybridization buffer/MABT (0.1 M maleic acid, 0.15 M NaCl, 0.1% Tween-20, pH 7.5) and further washed once for 15 min with MABT. The embryos were incubated with 1.5 ml of MABT/2% blocking reagent for 1 h and incubated for additional 1 h in 1.5 ml of MABT/2% blocking reagent/20% heat-treated sheep serum. Then, the embryos were incubated overnight at 4 °C in 1 ml of fresh MABT/2% blocking reagent/20% sheep serum containing 1:2,000 dilution of alkaline phosphatase (AP)-anti-DIG antibody. The embryos were washed five times for 4 h each and then overnight with 10–20 ml of MABT on a rocking incubator and washed three times for 60 min each with 10–20 ml of NTMT (0.1 M NaCl, 0.1 M Tris-HCl (pH9.5), 50 mM MgCl_2_ and 0.1% Tween-20). Finally, the embryos were incubated with AP substrate at 4 °C for 4 days until the background starts to come up. When colour had developed to the desired extent, the embryos were rinsed once and washed twice with PBS-T and refixed in 4% paraformaldehyde/0.1% glutaraldehyde/PBS-T for 2 h at room temperature. The embryos were rinsed once and washed two times for 10 min with PBS-T. The stained embryos were examined using an Olympus SZX16 stereo microscope equipped with a DP71 camera. Digoxigenin-labelled anti-sense RNA probes used in the present study were transcribed *in vitro* with T7 or Sp6 RNA polymerase as described by the manufacturer.

### Western blot analysis

Muscle tissues and C2C12 cells were lysed in a buffer containing 5 0 mM Tris (pH 7.5), 150 mM NaCl, 0.5% Nonidet P40, and protease and phosphatase inhibitors. Proteins in lysates were resolved by SDS–PAGE and transferred to a polyvinylidenedifluoride membrane. Immunoblotting was performed using primary antibodies against MHC (MF-20); MyoD (BD Biosciences); β-actin (Sigma); and Brg1 (Santa Cruz, sc-17796X). Anti-Baf60c antibody was a gift from Pier Lorenzo Puri. Membranes were washed for 30 min, incubated with HRP-conjugated secondary antibodies (Zhongshanjinqiao Corporation) for 1 h at room temperature, and then washed with Tris-buffered saline containing 0.1% Tween-20 for 30 min. Membranes were then placed in detection solution (Thermo Scientific), incubated for 1 min at room temperature, and subsequently exposed to X-ray film. Full scans of all Western blots are available in Supplementary Fig. 14.

### Statistical analysis

Results are presented as means±s.e's. Statistical significance of the difference between two means was calculated using Student's *t*-test. A *P*<0.05 was considered to represent a statistically significant difference.

### Data availability

The public RNA-seq data was downloaded from GEO (GSE20846). MyoD-regulated Linc-RNAs were identified using published ChIP-seq data downloaded from the SRA database using accession codes SRP001761 and SRA01085. The RNA-seq data gemerated in the present study have been deposited to GEO database (accession number: GSE72601).

## Additional information

**How to cite this article:** Yu, X. *et al*. Long non-coding RNA Linc-RAM enhances myogenic differentiation by interacting with MyoD. *Nat. Commun.* **8**, 14016 doi: 10.1038/ncomms14016 (2017).

**Publisher's note:** Springer Nature remains neutral with regard to jurisdictional claims in published maps and institutional affiliations.

## Supplementary Material

Supplementary Information Supplementary Figures 1–14 and Supplementary Table 1–3

Supplementary Dataset 1 264 genes are upregulated in linc-RAM overexpression C2C12 cells

Supplementary Dataset 2 Among 882 DE genes, 151 genes are regulated by MyoD
