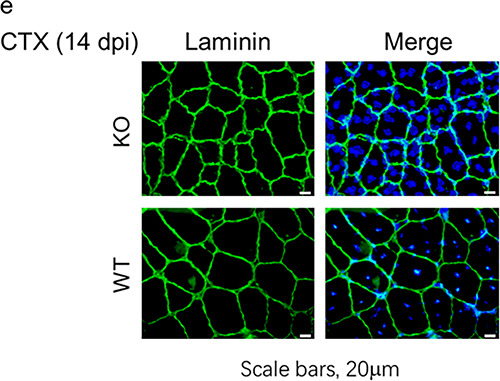# Author Correction: Long non-coding RNA Linc-RAM enhances myogenic differentiation by interacting with MyoD

**DOI:** 10.1038/s41467-025-64348-4

**Published:** 2025-12-16

**Authors:** Xiaohua Yu, Yong Zhang, Tingting Li, Zhao Ma, Haixue Jia, Qian Chen, Yixia Zhao, Lili Zhai, Ran Zhong, Changyin Li, Xiaoting Zou, Jiao Meng, Antony K. Chen, Pier Lorenzo Puri, Meihong Chen, Dahai Zhu

**Affiliations:** 1 The State Key Laboratory of Medical Molecular Biology, Institute of Basic Medical Sciences, Chinese Academy of Medical Sciences and Department of Biochemistry and Molecular Biology, School of Basic Medicine, Peking Union Medical College, Beijing, China; 2 Department of Biomedical Informatics, School of Basic Medical Sciences, Peking University Health Science Center, Beijing, China; 3 Department of Biomedical Engineering, College of Engineering, Peking University, Beijing, China; 4 Developmental Aging and Regeneration Program, Sanford-Burnham-Prebys Medical Discovery Institute, La Jolla, CA USA; 5 Department of Epigenetics and Regenerative Medicine, IRCCS Fondazione Santa Lucia, Rome, Italy

Correction to: *Nature Communications* 10.1038/ncomms14016, published online 16 January 2017

In the originally published version of the article, representative images of KO mice in Fig. 2e were inadvertently duplicated from WT samples. This was an error in presentation; the conclusions of the study remain unchanged. The panel has been updated using authentic KO mouse images, see Fig. 1 below. Due to the age of the paper, a direct replacement of the original figure is not possible; this amendment serves to update Fig. 2e.

Original Fig. 2e
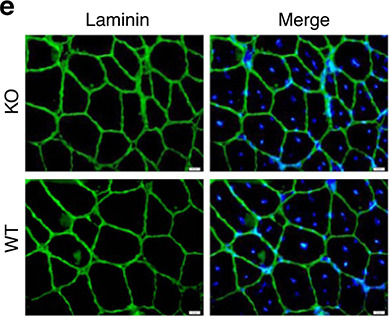


Revised Fig. 2e