# Supplementary material for: Long non-coding RNA Linc-RAM enhances myogenic differentiation by interacting with MyoD
Source: Nat Commun. 2017 Jan 16;8:14016. doi: 10.1038/ncomms14016 (PMC5241866; doi:10.1038/ncomms14016)
Supplement: Supplementary Information — Supplementary Figures 1–14 and Supplementary Table 1–3 [file ncomms14016-s1.pdf]

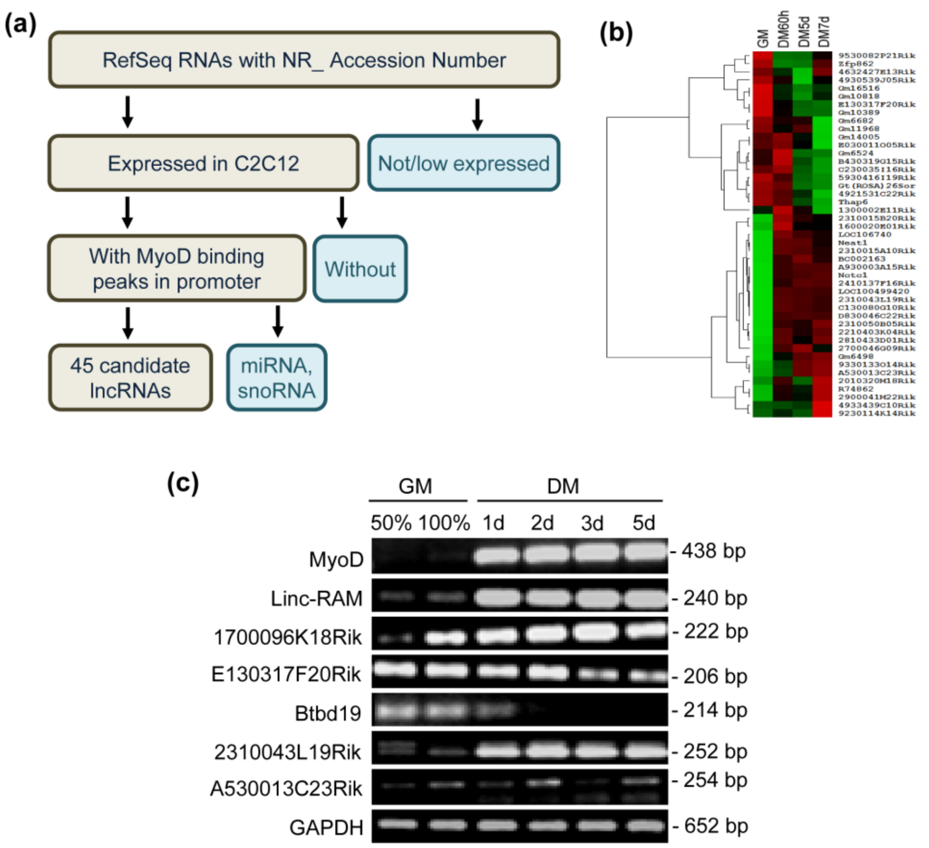

Supplementary Figure 1 | Systematic identification of muscle-expressed, MyoD-regulated lncRNAs. (a) The strategy for identifying lncRNAs that are potentially regulated by MyoD in muscle cells. **(b)** Heatmap representing hierarchical clustering of all genes that displayed a 1.5-fold difference at the transcriptional level between proliferation (GM) and differentiation (60 h, 5d, and 7d in DM). The 2310015B20Rik transcript is also known as NR_038041, termed Linc-RAM in the current study. **(c)** Six linc-RNA candidates in the top list were validated by semi-quantitative RT-PCR. Total RNA were isolated from C2C12 cells grown in either growth medium (GM) with different confluence (50% and 100%) or differentiation medium (DM) for different days (d).

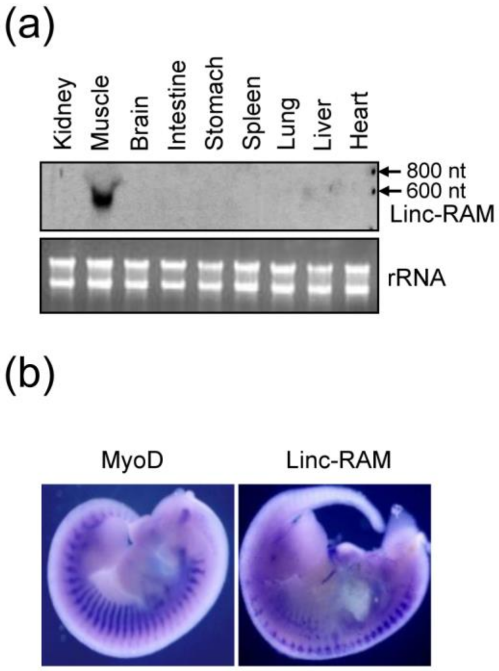

Supplementary Figure 2 | Linc-RAM is a muscle-specifically expressed lncRNA. (a) Expression of Linc-RAM in the indicated tissues was analyzed by Northern blotting. rRNA was used as a loading control. **(b)** Expression of Linc-RAM at the embryonic stage was detected by *in situ* hybridization in E11.5 mouse embryos. MyoD expression was used to demonstrate somite location.

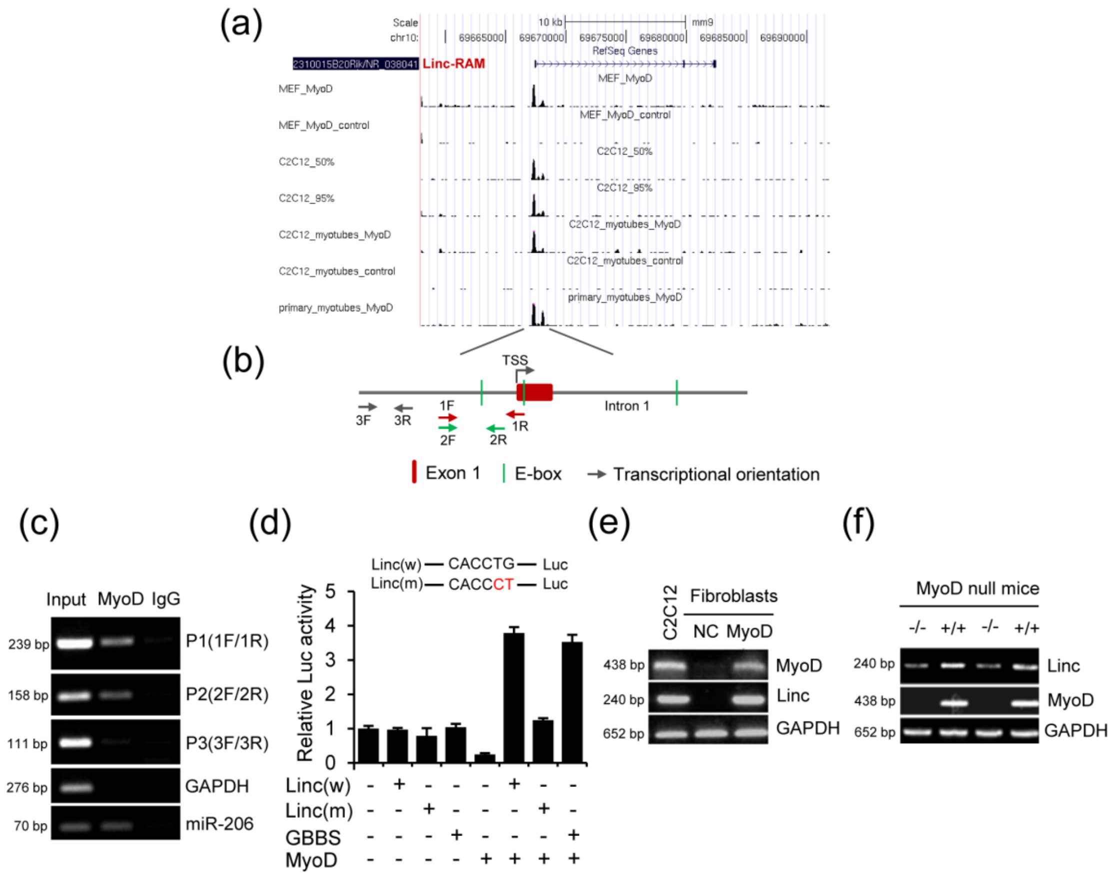

Supplementary Figure 3 | Linc-RAM is transcriptionally regulated by MyoD. (a) MyoD binding sites in the regulatory regions of the Linc-RAM gene were analyzed by using the published MyoD ChIP-Seq data (Cao et al.) **(b)** Identified E-box in the proximal promoter of Linc-RAM gene. The primers for ChIP-PCR analysis indicated as 1F/1R, 2F/2R, and 3F/3R. **(c)** ChIP assays were performed using chromatin from C2C12 cells with MyoD antibodies or control IgG. The precipitated DNA was amplified by real-time PCR with indicated primers P-1 and P-2 (covering the E-box of the *Linc-RAM* promoter) and the primers P3 (negative control covering a region in the *Linc-RAM* promoter lacking an E-box consensus sequence). *GAPDH* and *miR-206* promoter regions were also amplified as negative and positive controls, respectively. The data are representatives of three independent experiments. **(d)** Activities of *Linc-RAM* promoters containing a wild-type (Linc-W) or mutant (Linc-M) E-box were assayed by luciferase reporter assay in C3H-10T1/2 mouse embryo fibroblasts cotransfected with MyoD. The proximal promoter of the *MyoG* gene,

GBBS, a well-documented MyoD-responsive promoter, was used as positive control. The data are representatives of three independent experiments. **(e)** C3H-10T1/2 fibroblasts were transfected with MyoD to induce myogenic conversion, and Linc-RAM expression was subsequently detected by semi-quantitative RT-PCR. Myogenic C2C12 cells were used as a control for Linc-RAM expression. **(f)** Linc-RAM expression in the skeletal muscle of MyoD-knockout ($MyoD^{-/-}$) mice was examined by semi-quantitative RT-PCR. GAPDH served as a loading control.

(a)

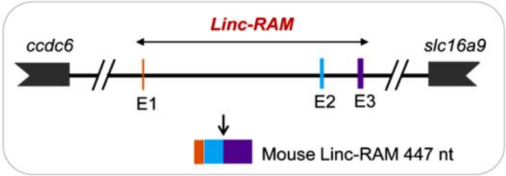

(b)

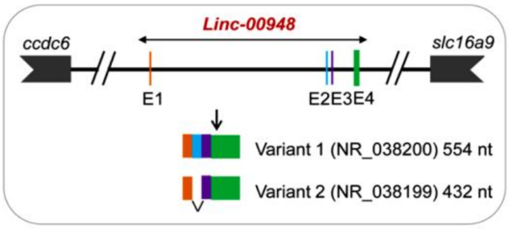

(c)

```
Mouse_Linc-RAM      CAGTGAGCTTTTCGTCCATGGAGAGCAGATGGTGTTTTTGA-AAGGAAGGGCGGCCATTC 59
Human_Linc-RAM_v1_  -----GCCTCCTGATCCACCAAGACCATATGGTGTTTTTCATAAAAGAGAGAGATCATTC 55
                           **  *  ****   *** ** *********** * **   ** * *  *****

Mouse_Linc-RAM      CCAGGACTTTGGACTTGGCTCCGTTGCACCCCTGAACA---GAACCAAC----GTTGCTA 112
Human_Linc-RAM_v1_  CCTTGACTTTGGACTTCGCTT-ATTGAACCCCTGGACAACGGAATCTCCCTCTGTCGCCC 114
                    **  ************ ***   *** ******* ***   *** *  *    **  **

Mouse_Linc-RAM      -----GGAGAACA-----------------------------------CCTGTGCCCCAGC 133
Human_Linc-RAM_v1_  AGGCTGGAGTGCAGTGGCACCATCTCGGCTTACCGCAACCTCCGCCTCCTGGGTTCAAAC 174
                         ****  **                                  **** *  * * *

Mouse_Linc-RAM      A---------CTTTGGTC-------------------CAG----CAAGG--GC-AGGAGA 158
Human_Linc-RAM_v1_  AATACTCCTACCTTGGCCTCCTGAGTAGCTGGGATTACAGGAATCAAGGTTGCTAAGAGA 234
                    *         * **** *                   ***    *****  ** * ****

Mouse_Linc-RAM      ---------------------------GG--AGAGCTGGAAACTGGTAGAGCTGTGCTAC 189
Human_Linc-RAM_v1_  CCACCTGTGCTGAATACACTTTGTTCTGGCAAGAGCAGAAAACTGAGA-AGCCGTGTTAC 293
                                               **  ***** * ******  * *** *** ***

Mouse_Linc-RAM      CTGCTACCTCCTGAGGCT--CTCAGGGAGCAGGAGAAT-CTGAACATGAGTGGCAAGAGC 246
Human_Linc-RAM_v1_  CTACCACTACCTGGGATTAATTCAGGGAGCGGGATAATTCTGGATATGACTGGTAAAAAC 353
                    ** * **  **** *  *   ********* *** *** *** * **** *** ** * *

Mouse_Linc-RAM      TGGGTACTGATCTCTACTACTTCGCCCCAGAGCCTGGAAGATGAGATTCTGGGACGGCTT 306
Human_Linc-RAM_v1_  TGGATATTAATTTCTACTACTACTCCCAAAAGTCTAGAAGATGAAATTGTGGGAAGACTT 413
                    *** ** * ** ********* * *** * ** ** ******** *** ***** * ***

Mouse_Linc-RAM      CTGAAAATTCTCTTCGTTTTGTTCGTTGACTTAATGTCCATTATGTATGTCGTCATAACG 366
Human_Linc-RAM_v1_  CTAAAAATTTTGTTTGTTATCTTTGTTGACTTAATTTCTATTATATATGTTGTGATAACT 473
                    ** ****** * ** *** * ** *********** ** ***** ***** ** *****

Mouse_Linc-RAM      TCCTAGCAACCTGACTTTCTTTACTCCGTGTGTAAGTTTCAAACCAAATGCGAGCAAATA 426
Human_Linc-RAM_v1_  TCTTAGAAAGCTGACTTCCTTT---CCATGTACA--TTTCAAACTGAATTACAGTGAATA 528
                    ** *** ** ******* ****   ** ***  *  ********  ***   **  ****

Mouse_Linc-RAM      AAGATCTACATTTTAATCTGA----- 447
Human_Linc-RAM_v1_  AAAATTTGTATTTTAACTTGTAAAAA 554
                    ** ** *  *******  **
```

(d)

```
Mouse_Linc-RAM      CAGTGAGCTTTTCGTCCATGGAGAGCAGATGGTGTTTTTGA-AAGGAAGGGCGGCCATTC 59
Human_Linc-RAM_v2_  -----GCCTCCTGATCCACCAAGACCATATGGTGTTTTTCATAAAAGAGAGAGATCATTC 55
                           **  *  ****   *** ** *********** * **   ** * *  *****

Mouse_Linc-RAM      CCAGGACTTTGGACTTGGCTCCGTTGCACCCCTGAACAGAACCAACGTTGCTAGGAGAAC 119
Human_Linc-RAM_v2_  CCTTGACTTTGGACTTCGCTT-ATTGAACCCCTGGACAGAATCAAGGTTGCTAAGAGACC 114
                    **  ************ ***   *** ******* ****** *** ******* **** *

Mouse_Linc-RAM      ACCTGTGCCCCA-GCACTTTGGTCCAGCAAGGGCAGGAGAGGAGAGCTGGAAACTGGTAG 178
Human_Linc-RAM_v2_  ACCTGTGCTGAATACACTTTGTTCTGGCAAGAGCAG-------------AAAACTGAGA- 160
                    ********   *  ******* **  ***** ****              ******  *

Mouse_Linc-RAM      AGCTGTGCTACCTGCTACCTCCTGAGGCT--CTCAGGGAGCAGGAGAAT-CTGAACATGA 235
Human_Linc-RAM_v2_  AGCCGTGTTACCTACCACTACCTGGGATTAATTCAGGGAGCGGGATAATTCTGGATATGA 220
                    *** *** ***** * **  **** *  *   ********* *** *** *** * ****

Mouse_Linc-RAM      GTGGCAAGAGCTGGGTACTGATCTCTACTACTTCGCCCCAGAGCCTGGAAGATGAGATTC 295
Human_Linc-RAM_v2_  CTGGTAAAAACTGGATATTAATTTCTACTACTACTCCCAAAAGTCTAGAAGATGAAATTG 280
                     *** ** * **** ** * ** ********* * *** * ** ** ******** ***

Mouse_Linc-RAM      TGGGACGGCTTCTGAAAATTCTCTTCGTTTTGTTCGTTGACTTAATGTCCATTATGTATG 355
Human_Linc-RAM_v2_  TGGGAAGACTTCTAAAAATTTTGTTTGTTATCTTTGTTGACTTAATTTCTATTATATATG 340
                    ***** * ***** ****** * ** *** * ** *********** ** ***** ****

Mouse_Linc-RAM      TCGTCATAACGTCCTAGCAACCTGACTTTCTTTACTCCGTGTGTAAGTTTCAAACCAAAT 415
Human_Linc-RAM_v2_  TTGTGATAACTTCTTAGAAAGCTGACTTCCTTT---CCATGTACA--TTTCAAACTGAAT 395
                    * ** ***** ** *** ** ******* ****   ** ***  *  ********  ***

Mouse_Linc-RAM      GCGAGCAAATAAAGATCTACATTTTAATCTGA----- 447
Human_Linc-RAM_v2_  TACAGTGAATAAAAATTTGTATTTTAACTTGTAAAAA 432
                       **  ****** ** *  *******  **
```

Supplementary Figure 4 | Schematic representations of the flanking genes and genomic structure of mouse Linc-RAM and its human homolog. (a) The upstream of mouse Linc-RAM is *Ccdc6* (coiled-coil domain containing 6) and the downstream gene is *Slc16a9* (solute carrier family 16, member 9). **(b)** Human Linc-00948 and mouse Linc-RAM located at the same syntenic region, suggesting Linc-00948 is a human homolog for mouse Linc-RAM. Exons of Linc-RAM and Linc-00948 are labeled E1, E2, E3, and E4. **(c, d)** Sequence similarity between human Linc-00948 and mouse Linc-RAM

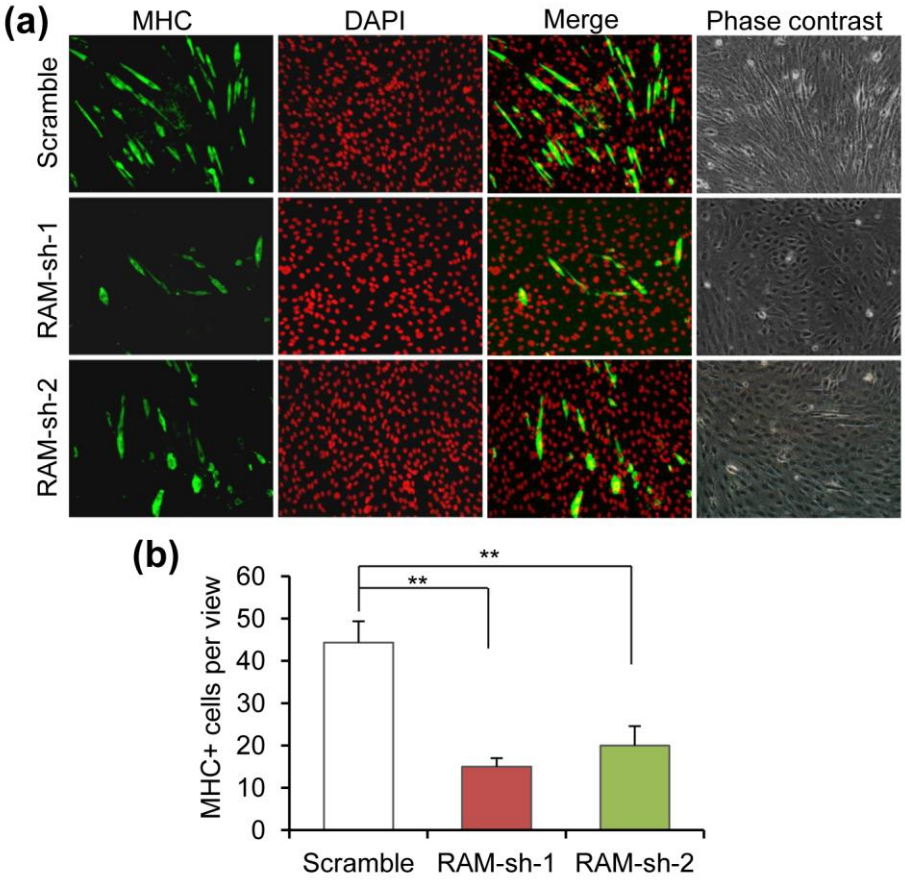

Supplementary Figure 5 | knocking down of Linc-RAM with two independent of sh-RAM significantly attenuate C2C12 cell differentiation. (A) Representative images of MHC immunostaining on C2C12 cells transiently transfected with RAM-sh-1 and RAM-sh-2 in DM for 48 hours. Scramble sequence were used as negative control. (B) MHC+ cell numbers were calculated based on MHC immunostaining in panel A. The data are presented as mean ± s.e.m from three independent experiments. The statistical significance was calculated with the t test. $^{**}P < 0.01$.

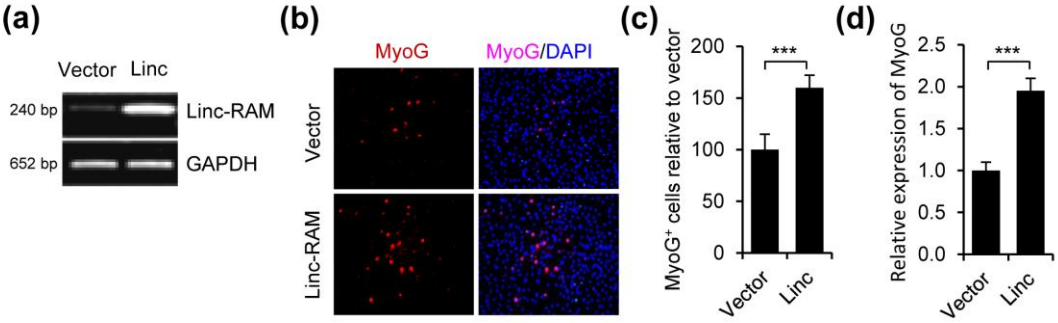

Supplementary Figure 6 | Linc-RAM promotes C2C12 cell differentiation. (**a**) C2C12 cells were transiently transfected with full-length Linc-RAM and harvested after culturing in differentiation medium (DM) for 24 hours. Overexpression of full-length Linc-RAM was examined by semi-quantitative RT-PCR. (**b**) Differentiation of C2C12 cells transfected with full-length Linc-RAM was examined by staining for MyoG after culturing in DM for 24 hours. (**c**) $MyoG^+$ cells in (**b**) were counted and presented as a percentage relative to the negative control (NC) group (defined as 100%). (**d**) MyoG mRNA expression in (**b**) was detected by RT-qPCR. All images in the figure are representatives of three independent experiments. Values are means ± s.e.m of three independent experiments. The statistical significance of the difference between two means was calculated with the *t* test. ***$P < 0.001$.

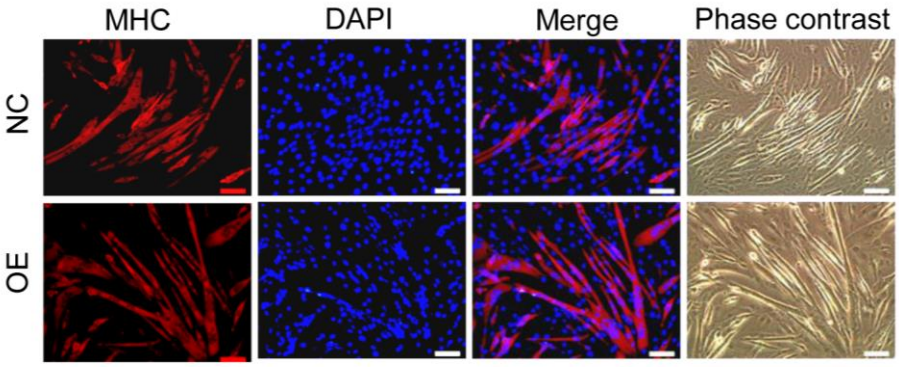

Supplementary Figure 7 | Linc-RAM enhances C2C12 cell differentiation and fusion. The C2C12 cells overexpressing Linc-RAM were differentiated for 48h in differentiation medium. MHC immunostained myotubes and the phase contrast photos were presented. Scale bar, 50μm.

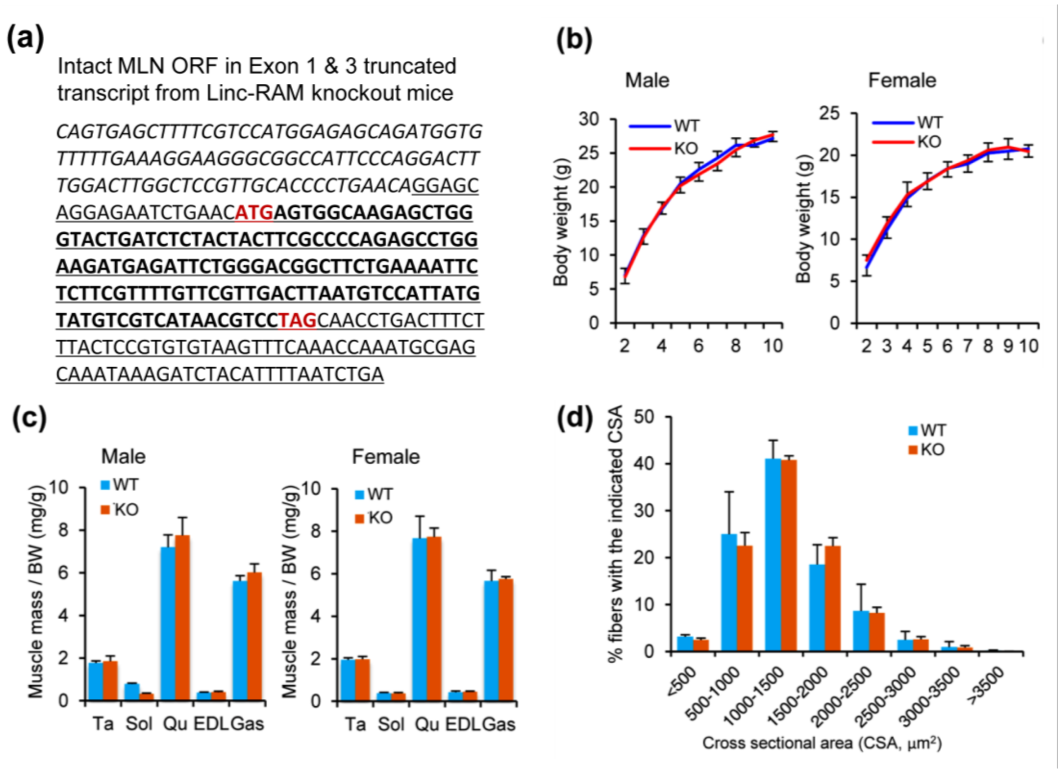

Supplementary Figure 8 | Linc-RAM knockout mice do not exhibit overt abnormalities. (**a**) Strategy for generation of Linc-RAM knockout mice. (**b**) Body weight of Linc-RAM-/- (KO) and their wild type littermates (WT). 10 mice were weighted for each group in both genders. Values are means ± s.e.m. (**c**) Muscle mass of Linc-RAM-/- (KO) and their wild type littermates (WT). Ta is *tibialis anterior*, Sol is *soleus*, EDL is *extensor digitorum longus*, Qu is *quadriceps*, and Gas is *gastrocnemius* muscle, respectively. Values are mean ± s.e.m. n = 8 mice for each group in both genders. (**d**) Cross-sectional area of myofibers in Ta muscle from the WT and KO mice. n = 7 mice of each genotype. Values are means ± s.e.m.

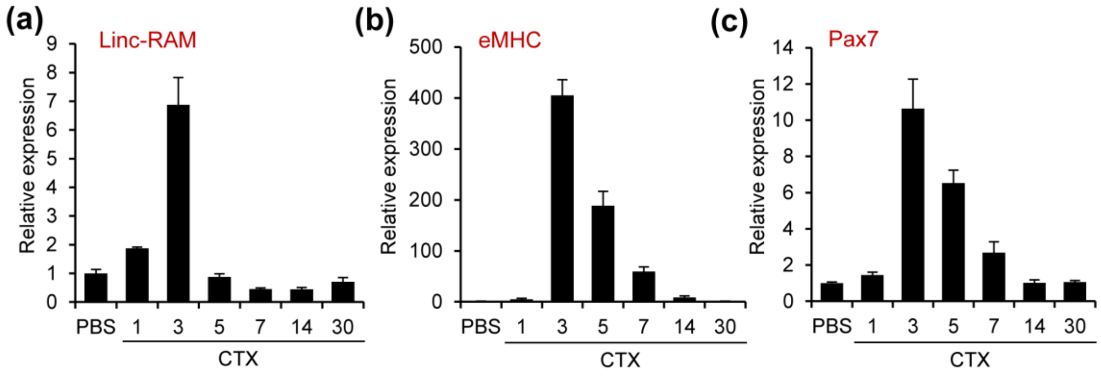

Supplementary Figure 9 | Expression pattern of Linc-RAM during CTX-induced muscle injury and regeneration. Muscle injury was induced by injecting CTX into the right TA muscle of C57BL/6 mice (n=5). PBS injection into the left TA muscle was the control. Damaged muscles were harvested on the indicated days after CTX injury. (**a**–**c**) Expression of *Linc-RAM* (**a**), *eMHC* (**b**), and *Pax7* (**c**) were determined by real-time RT-PCR. *GAPDH* was the internal control.

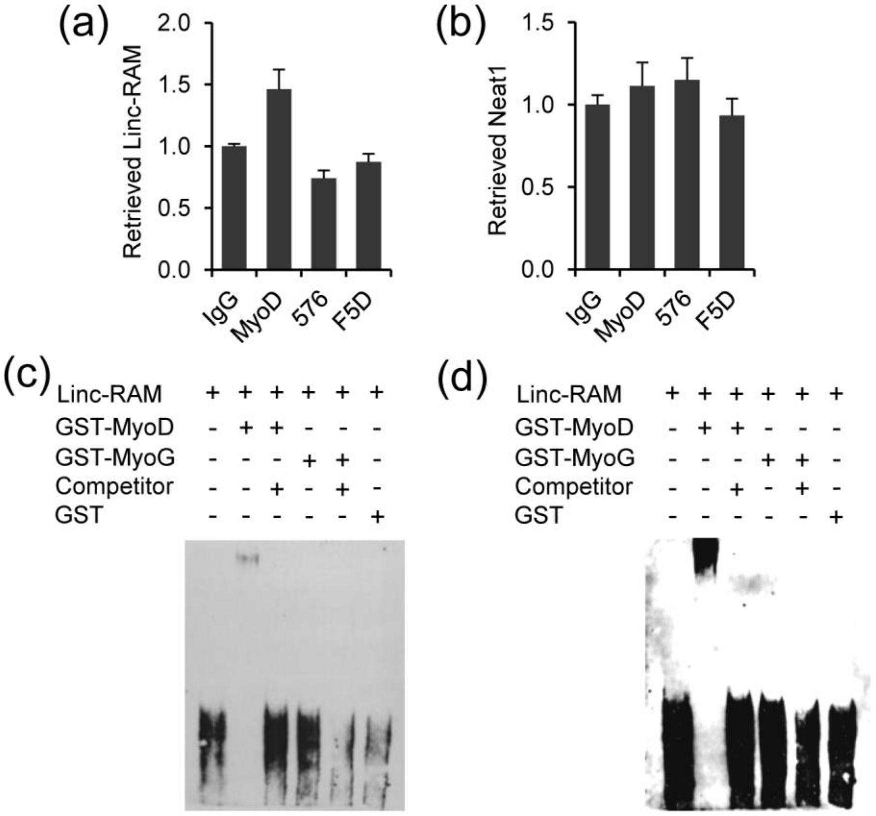

Supplementary Figure 10 | Linc-RAM does not bind MyoG. (a) C2C12 cell lysates were respectively immunoprecipitated using two anti-MyoG (sc576) and anti-MyoG (F5D). Subsequently, Linc-RAM in the immunoprecipitates was detected by real-time RT-qPCR. IgG and MyoD antibodies served as a negative and positive controls, respectively. **(b)** Neat1 as a negative control in the immunoprecipitates described in panel a was detected by real-time RT-qPCR. **(c)** The interaction between Linc-RAM and MyoG was examined by electrophoresis mobility shift assay (EMSA) with purified recombinant GST-MyoG fusion protein. GST-MyoD served as a positive control and GST only served as a negative control. Linc-RAM RNA without Dig-UTP-labeling served as competitor RNA. The presented blot was a representative of three independent experiments. **(d)** Long exposure of the blot presented in panel c of this Figure.

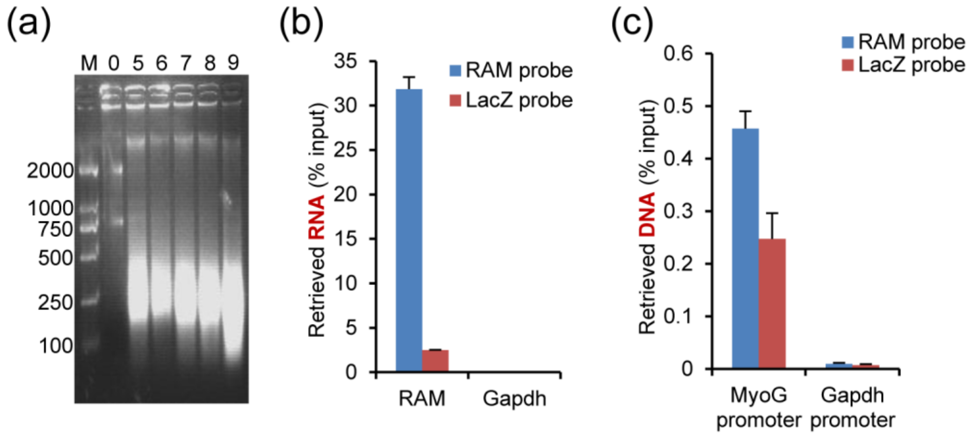

Supplementary Figure 11 | ChIRP analysis demonstrates Linc-RAM binds myogenin gene promoter. (a) Sonicated chromatin was separated on 1.5% agarose gel. The C2C12 cells grown in differentiation medium for 24h were crosslinked with 1% glutaraldehyde and subsequently sonicated for 9 cycles with program (30 seconds sonication, 30 seconds interval, repeat 10 times). M: molecular marker. 0: non-sonicated samples. 5~9: the samples were sonicated for 5~9 cycles, respectively. **(b)** The retrieved RNA by Linc-RAM or LacZ probe from ChIRP experiment was analyzed by real-time RT-PCR with primers against Linc-RAM RNA. Gapdh served as negative control for ChIRP analysis. **(c)** The retrieved genomic DNA by Linc-RAM or LacZ probe from ChIRP experiment was analyzed by real-time PCR with primers against myogenin gene promoter. Gapdh served as negative control.

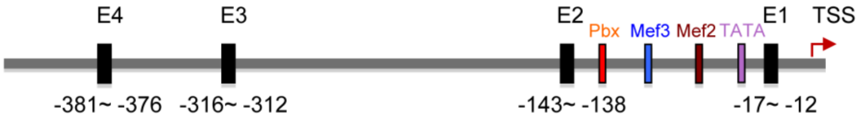

Supplementary Figure 12 | Schematic illustration of the *MyoG* gene promoter.

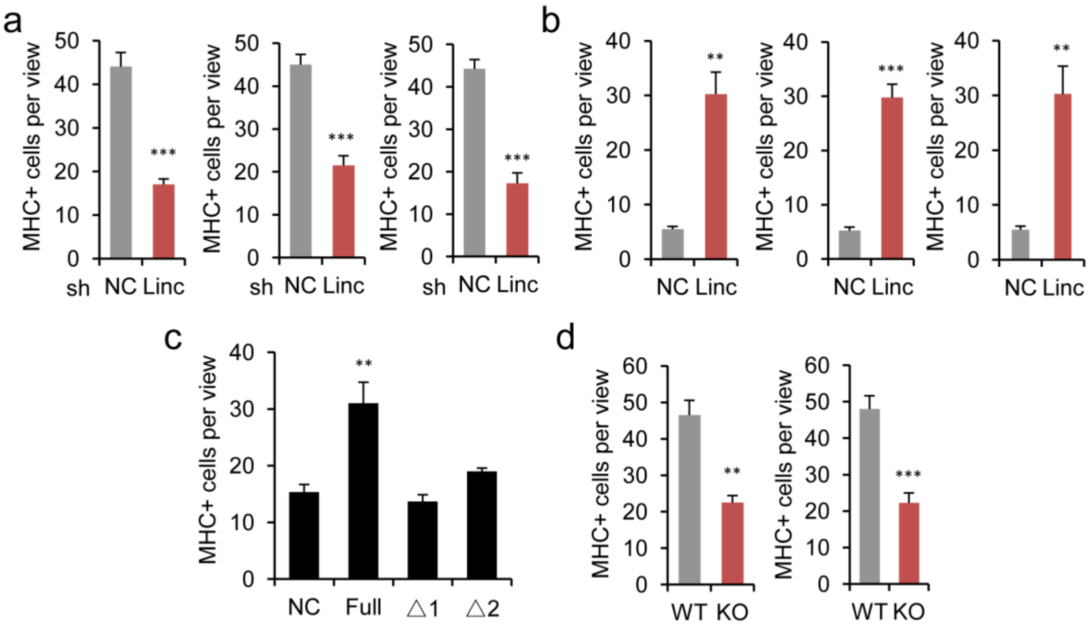

Supplementary Figure 13 | MHC positive cells calculated in three or two independent experiments. (**a**) Individual data from three independent experiments which were related to Figure 1b and 1c in main text. (**b**) Individual data from three independent experiments which were related to Figure 1f and 1g in main text. (**c**) The data were from another independent experiment which were related to Figure 1j and 1k in main text. (**d**) Individual data from additional two independent experiments which were related to Figure 2f and 2g in main text.

Original blots

For Fig. 1d

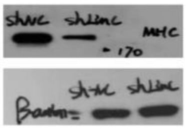

For Fig. 1h

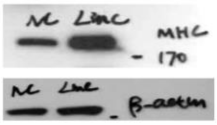

For Fig. 3d

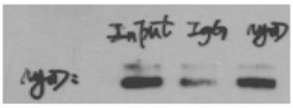

For Fig. 5d

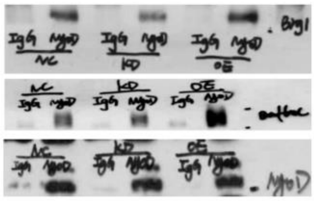

For Fig. 5h

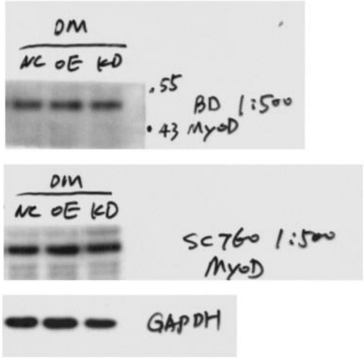

For Supplementary Figure 2

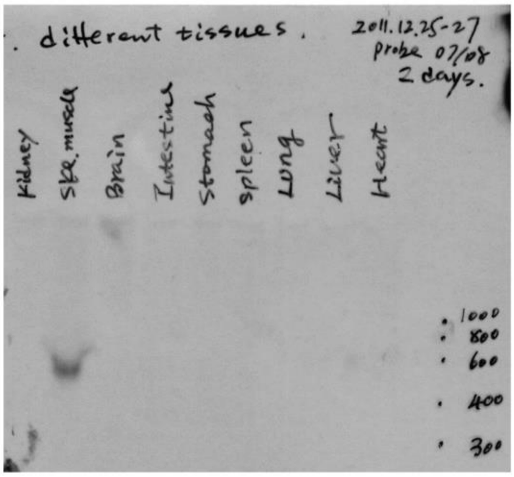

Supplementary Figure 14 | Original blots for the Figures in main text

Supplementary Table 1 Selected genes that are coregulated by Linc-RAM and MyoD from main text Figure 4c.

Growth differentiation factor 15 (*Gdf15*), Cadherin 15 (*Cdh15*), Collagen, type IV, alpha 1 (*Col4a1*), Junction adhesion molecule 2 (*Jam2*), Junctional sarcoplasmic reticulum protein 1 (*Jsrp1*), Integrin alpha 7 (*Itga7*), transmembrane protein 8C (Tmem8c), Cholinergic receptor, nicotinic, delta polypeptide (*Chrnd*).

Function	Symbol	Fold
Differentiation		
	Myod1	2.0
	Myog	5.6
	Gdf15	4.6
Cell adhension and junction		
	Cdh15	1.8
	Col4a1	1.5
	Jam2	2.8
	Jsrp1	3.7
	Itga7	3.3
	Tmem8c	1.9
	Chrnd	2.5

Supplementary Table 2 All primers used in the study

(1) Primers used in Real-time RT-PCR experiments

**Gene**	**primers**
Tmem8c-FW	ATCGCTACCAAGAGGCGTT
Tmem8c-RV	CACAGCACAGACAAACCAGG
MyoG-FW	GCAATGCACTGGAGTTCGGT
MyoG-RV	GCTGTCCACGATGGACGTAAG
MyoD-FW	CAACGCCATCCGCTACAT
MyoD-RV	GGTCTGGGTTCCCTGTTCT
Col4a1-FW	CAGGACAAAAGGGTGATGCT
Col4a1-RV	CCTTTGTACCGTTGCATCCT
Linc-RAM-RW	GGCGGCCATTCCCAGGACTTTG
Linc-RAM-RV	ATCTTCCAGGCTCTGGGGCGAAG
GAPDH-FW	TGGAGAAACCTGCCAAGTATGA
GAPDH-RV	CTGTTGAAGTCGCAGGAGACA
Baf60c-FW	AGGGGAAGCTCTTGGATGAT
Baf60c-RV	CCATCTGTTTCCTGGGTTGT
Brg1-FW	CTGCGTAAGATCTGCAACCA
Brg1-RV	TTGGCAAAAGAGGAGCACTT
MHC-FW	GATGGCACCGAAGTTGCTG
MHC-RV	TACTCATTGCCGACCTTGACC
miR-133b-FW	TTTGGTCCCCTTCAACCAGCTA
miR-133-RV	GTCGTATCCAGTGCAGGGTCCGAGGTATTCGCACT GGATACGACTAGCTG
miR-206-FW	TGGAATGTAAGGAAGTGTGTGG
miR-206-RV	GTCGTATCCAGTGCAGGGTCCGAGGTATTCGCACT GGATACGACCCACAC
Common Reverse primer for miR	GTGCAGGGTCCGAGGT
Pax-7-FW	CCGTGTTTCTCATGGTTGTG
Pax-7-RV	GAGCACTCGGCTAATCGAAC
eMHC-FW	TCTAGCCGGATGGTGGTCC
eMHC-RV	GAATTGTCAGGAGCCACGAAA
Neat1-FW	GGGAAGGGTGACATTGAAAA
Neat1-RV	CTCCCCAGCTTCACTTCTTG

(2) Primers used in semi-quantitative RT-PCR

**Gene**	**primers**
GAPDG-FW	AATGTGTCCGTCGTGGATCTG
GAPDH-RV	TAGCCCAAGATGCCCTTCAGT
MyoD-FW	CAACGCCATCCGCTACAT

MyoD-RV	GGTCTGGGTTCCCTGTTCT
Neat1-FW	GGGAAGGGTGACATTGAAAA
Neat1-RV	CTCCCCAGCTTCACTTCTTG
1700096K18Rik-FW	CAGCGCACTACTTGTCGCGGA
1700096K18Rik-RV	CCCCGCCGTGGTCCCCTAAG
E130317F20Rik-FW	CGGTCGCCCTAAGCCAACCG
E130317F20Rik-RV	GACGGGTAAGTCCTGCCGCG
Btbd19-FW	CGTGGGCGCCGTTGAACAGA
Btbd19-RV	CCAACGCAGAGCACCTCGGG
2310043L19Rik-FW	GCACCTTGAGTGGTGACAGGGC
2310043L19Rik-RV	ACACAGAGGTGGGCCTCAACGT
A530013C23Rik-FW	CCCAGGGGCAACGCTTCACG
A530013C23Rik-RV	AGCTCCGCGCTGTTTGAGCAA

(3) Primers used in ChIP experiments

P1-FW (linc-RAM)	CTCCCTGCAGAAAAGGAGTG
P1-RV (linc-RAM)	CCGAGTGTTAGGCAGAAACC
P2-FW (linc-RAM)	AGGCAAGCAAACAGGCTAGA
P2-RV (linc-RAM)	GGGTGTTTAAGGAGGGCAAT
P3-FW (linc-RAM)	CTCCCTGCAGAAAAGGAGTG
P3-RV (linc-RAM)	TGCCTTAAGTCCCCATGGTA
MyoG promoter-FW	GAATCACATGTAATCCACTGGA
MyoG promoter-RV	ACGCCAACTGCTGGGTGCCA
miR-206 promoter-FW	GGAGTGATTGAGGTGGACAGA
miR-206 promoter-RV	CTCTCCCACCTGTTTGTGTCTT
GAPDH promoter-FW	AAGCCAAACTAGCAGCTAGG
GAPDH promoter-RV	GGGCTAGTCTATCATTGCAG

(4) Primers for plasmid construction

Privus-RAM-OE-FW	GGAATTCCCAGTGAGCTTTTCGTCCATGG
Privus-RAM-OE-RV	ATAAGAATGCGGCCGCTAAACTATTCAGATTAAAATG TAGATCT
Privus-Δ1-FW	GGAATTCCCAGTGAGCTTTTCGTCCATGG
Privus-Δ1-RV	ATAAGAATGCGGCCGCTAAACTATCTGAGAGCCTCAG GAGGTAG
Privus-Δ2-FW	GGAATTCCGAACCAACGTTGCTAGGAGA
Privus-Δ2-RV	ATAAGAATGCGGCCGCTAAACTATTCAGATTAAAATG TAGATCT

Lv-RAM-OE-full-FW	GGAATTCCCAGTGAGCTTTTCGTCCATGG
Lv-RAM-OE-full-RV	ATAAGAATGCGGCCGCTAAACTATTCAGATTAAAATGTAGATCT
MyoG promoter-FW	CGACGCGTCGGCTGAGCAGGAAAGAGAAGG
MyoG promoter-RV	GAAGATCTTCATAGAAGTGGGGCTCCTGGT
RAM promoyer-FW	GGGGTACCCCCTCCAGCAGGCCTGATATTC
RAM promoyer-RV	CCCAAGCTTGGGGAGCCAAGTCCAAAGTCCTG
MyoD overexpressing-FW	GGAATTCCGACAGGGAGGAGGGGTAGAG
MyoD overexpressing-RV	AAGGAAAAAAGCGGCCGCAAAAGGAAAAAGGGCTCCAGAAAGTGACAA

Supplementary Table 3 Probes for RNA-FISH and ChIRP analysis

(1) The set of probes against the Linc-RAM RNA sequence used for FISH imaging. All of the probes were labeled with a Cal 610 Fluor® Red (Cal610) flurophore at the 3’end.

1	ctgctctccatggacgaa
2	cgcccttcctttcaaaaa
3	ccaaagtcctgggaatgg
4	gggtgcaacggagccaag
5	gcaacgttggttctgttc
6	tggggcacaggtgttctc
7	ccttgctggaccaaagtg
8	agtttccagctctcctct
9	caggtagcacagctctac
10	tgagagcctcaggaggta
11	gttcagattctcctgctc
12	atcagtacccagctcttg
13	ctggggcgaagtagtaga
14	gaatctcatcttccaggc
15	attttcagaagccgtccc
16	gtcaggttgctaggacgt
17	tgctcgcatttggtttga

(2) Probes for Linc-RAM ChIRP analysis were labeled with BiotinTEG at the 3’end

#	Probe (5'-> 3')
RAM1	ccatggacgaaaagctcact
RAM2	aaaaacaccatctgctctcc
RAM3	aagtccaaagtcctgggaat
RAM4	ttctgttcaggggtgcaacg
RAM5	acaggtgttctcctagcaac
RAM6	cttgctggaccaaagtgctg
RAM7	tagcacagctctaccagttt
RAM8	cactcatgttcagattctcc
RAM9	agtagtagagatcagtaccc
RAM10	agaatctcatcttccaggct
RAM11	agaattttcagaagccgtcc
RAM12	ggacgttatgacgacataca
RAM13	ggtttgaaacttacacacgg
